# Integrative Regulatory Networks of MicroRNA-483: Unveiling Its Systematic Role in Human Diseases and Clinical Implications

**DOI:** 10.3390/biom15121707

**Published:** 2025-12-07

**Authors:** Jiatong Xu, Shupeng Luxu, Hsi-Yuan Huang, Yang-Chi-Dung Lin, Hsien-Da Huang

**Affiliations:** 1 School of Medicine, The Chinese University of Hong Kong, Shenzhen 518172, China; 2 Warshel Institute for Computational Biology, School of Medicine, The Chinese University of Hong Kong, Shenzhen 518172, China; 3 Guangdong Provincial Key Laboratory of Digital Biology and Drug Development, The Chinese University of Hong Kong, Shenzhen 518172, China; 4 Department of Endocrinology, Key Laboratory of Endocrinology of National Health Commission, Peking Union Medical College Hospital, Chinese Academy of Medical Sciences & Peking Union Medical College, Beijing 100730, China

**Keywords:** miRNA-483, regulatory network, ceRNA, transcription factor, biomarker, therapeutic target

## Abstract

MicroRNA-483 regulates multiple human disease categories, spanning oncology, cardiopulmonary, metabolic, immune, neurological, and musculoskeletal pathologies. We integrate experimentally validated interactions from 146 studies to construct a comprehensive regulatory network, encompassing transcription factors, long non-coding RNAs, circular RNAs, and messenger RNA targets. Our analysis reveals that miR-483 promotes tumorigenesis by suppressing tumor-suppressive checkpoints, yet it protects cardiopulmonary, metabolic, and neurological tissues from pathological injury. This functional duality arises from tissue-specific modulation of shared signaling pathways, particularly TGF-β and MAPK cascades, which function as the core hubs driving its context-dependent activity across six disease categories. By mapping miR-483 regulatory circuits across multiple diseases, we define the molecular determinants of its context-dependent activity. These findings establish miR-483 as both a diagnostic biomarker and a therapeutic target whose function is dictated by cellular context.

## 1. Introduction

MicroRNAs (miRNAs) are small endogenous non-coding RNAs of approximately 22 nucleotides that play critical roles in post-transcriptional gene regulation [1]. By binding to the 3′-untranslated regions (3′-UTR) of target messenger RNAs (mRNAs), miRNAs can cause mRNA degradation or block translation, strongly influencing gene expression and biological processes [1,2]. Aberrant miRNA expression has been closely linked to the progression of diverse human diseases, including cancers, metabolic disorders, and cardiovascular diseases, primarily through miRNA–target interactions (MTIs) [3]. Extensive databases such as miRTarBase [4] and TarBase [5] have cataloged thousands of experimentally validated MTIs, underscoring miRNA regulatory networks’ complexity and clinical significance.

Among the extensive repertoire of human miRNAs, microRNA-483 (miR-483) has emerged as a particularly pivotal regulator due to its pervasive dysregulation across a broad spectrum of human pathologies, from malignancies [6,7,8,9,10,11,12,13,14,15] to metabolic [16,17,18,19] and cardiovascular disorders [20,21,22,23,24,25,26,27]. Our previous work has specifically characterized its role in cardiovascular diseases, revealing its involvement in atherosclerosis susceptibility [28]. Its distinctive genomic architecture within the *IGF2* locus couples its expression with fundamental developmental pathways [29], while its detection in circulation has established it as a promising biomarker for early cancer diagnosis and aging assessment [30,31,32,33]. Therefore, this review aims to synthesize current knowledge of miR-483 as a paradigm for miRNA multifunctionality, providing insights applicable to the broader field of regulatory RNA biology.

**Figure 1:**
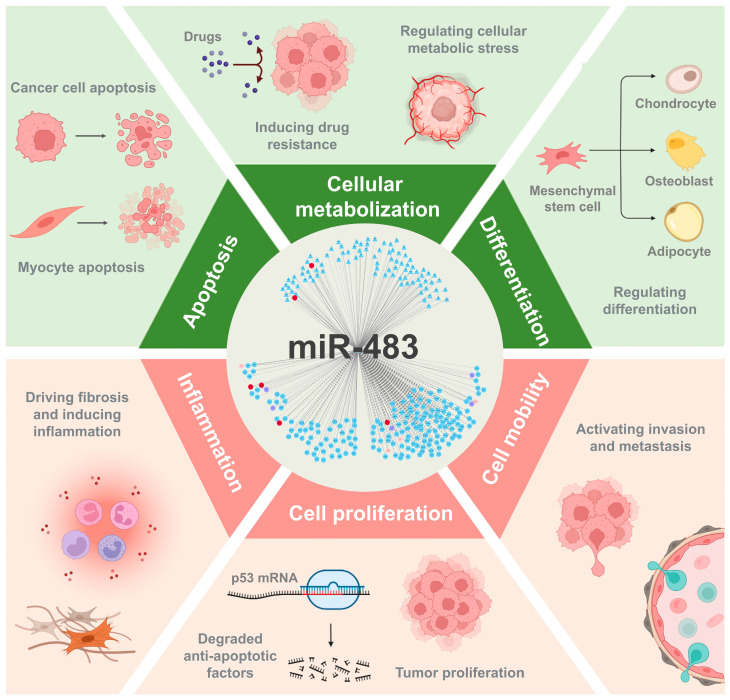
The Multifaceted Functions of miR-483. This diagram illustrates how miR-483 acts as a central regulatory hub, influencing diverse cellular processes by mediating its effects through interactions with upstream transcription factors and downstream mRNA targets. These interactions allow miR-483 to modulate key biological outcomes, including promoting tumor proliferation and invasion, regulating the differentiation of mesenchymal stem cells, controlling apoptosis in various cell types, and driving inflammation and fibrosis. This figure was created in Biorender Xu, J. (2025) https://BioRender.com/pyuku5w (accessed on 1 December 2025).

Genomically, miR-483 is localized within the intron of the insulin-like growth factor 2 (*IGF2*) gene [34]. The precursor miR-483 is processed into two mature isoforms: miR-483-5p, derived from the 5′ arm (5′-AAGACGGGGAGAGAGAGGAGACUU-3′), and miR-483-3p, from the 3′ arm (5′-UCACUCCUCCCCUCCCGUCUU-3′) [35]. Their distinct seed sequences confer unique target specificities, leading to differential regulatory roles in various biological processes and diseases. Both isoforms of miR-483 can significantly impact cellular metabolism [36], proliferation [37], differentiation [38], mobility [39], and apoptosis [40] through direct interactions with various critical targets, playing essential roles in diverse disease contexts (Figure 1).

The dysregulation of miR-483 is primarily controlled by its upstream regulatory mechanisms. As an intragenic miRNA, miR-483 is primarily co-transcribed with its host gene *IGF2* from paternal alleles due to genomic imprinting, which typically ensures paternal-specific expression and silencing of maternal alleles via imprinting control regions [41]. Aberrations in imprinting at the *IGF2*/*H19* locus frequently result in loss of imprinting, leading to biallelic expression of *IGF2* and consequently elevated levels of miR-483 [42]. Moreover, the co-regulation of miR-483 with *IGF2* mediated by transcriptional repressors such as ZBED6 and WT1 has been implicated in developmental processes like muscle growth and pathological conditions, including tumorigenesis [43]. However, the expression of miR-483 can also be independently modulated through various pathways, including Wnt/β-catenin signaling [44], inflammation-induced transcription factors (TFs) [41], as well as through competitive endogenous RNA (ceRNA) interactions involving long non-coding RNAs (lncRNAs) [45] and circular RNAs (circRNAs) [46]. These distinct regulatory modes underscore the complexity of miR-483 regulation, involving both *IGF2*-dependent and *IGF2*-independent mechanisms.

Despite considerable research into individual regulatory mechanisms within these diseases, a comprehensive systematic integrative analysis of miR-483-mediated networks across multiple disease contexts remains lacking. To address this gap, this review systematically integrates existing knowledge regarding miR-483’s upstream regulators, including TFs, lncRNAs, and circRNAs, and downstream target interactions. We subsequently construct integrative miR-483-mediated regulatory networks, highlighting core conserved pathways such as epithelial–mesenchymal transition (EMT), TGF-β, MAPK, and IGF signaling while delineating disease-specific regulatory characteristics. Finally, we discuss miR-483’s clinical implications as a promising diagnostic biomarker and potential therapeutic target, aiming to provide valuable insights and facilitate future research and translational applications.

## 2. Upstream Regulators of miR-483

Various upstream regulatory elements, including TFs, circRNAs, and lncRNAs, influence miRNAs’ expression. These regulators modulate miRNA expression directly or indirectly, affecting the downstream target gene interactions and functional roles of miRNAs. Elucidating the upstream regulatory mechanisms of miR-483 is essential for a comprehensive understanding of its multifaceted involvement in human diseases (Table 1).

### 2.1. TF-miR-483 Regulation

**Figure 2:**
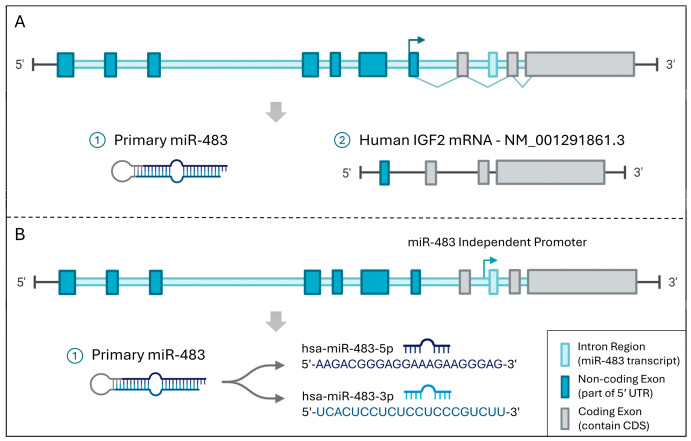
The schematic illustrates the location of miR-483 within the intron of the *IGF2* gene chromosome 11 (GRCh38.p14) [80]. (**A**) Co-transcribed with host gene: Transcription is driven by the upstream *IGF2* promoter (dark blue arrow), resulting in the co-expression of the *IGF2* mRNA and the primary miR-483 transcript by exon junction. (**B**) miRNA independent transcription: Transcription is driven by an internal miR-483-specific promoter located within the intron (light blue arrow). This produces primary miR-483, which is subsequently processed into mature hsa-miR-483-5p and hsa-miR-483-3p. The *IGF2* gene consists of 10 exons, dark blue boxes represent non-coding exons that are transcribed into mRNA but not translated into protein; gray boxes represent coding exons contain the coding sequence (CDS) that actually gets translated into protein; the light blue regions denote introns, harboring the miR-483 sequence [77].

Transcription factors (TFs) play pivotal roles in modulating miRNA expressions at the transcriptional level through distinct regulatory patterns that can be categorized as co-regulation or independent regulation [76]. For miR-483, these regulatory patterns reflect the complex genomic architecture of its intronic location within the *IGF2* gene on chromosome 11 [77] (Figure 2). Several TFs have been identified as direct regulators of miR-483. The β-catenin (CTNNB1)/upstream stimulatory factor 1 (USF1) complex has been validated as a key activator of miR-483-3p transcription, multiple studies showed that β-catenin interacts with USF1 to drive miR-483 expression independently of its host gene *IGF2*, linking this regulation to Wnt/β-catenin signaling and enhancing oncogenic functions such as inhibition of *TP53*-mediated apoptosis [42,44,47]. Under high-glucose conditions, O-linked N-acetylglucosamine transferase (OGT) further promotes this activation by stabilizing β-catenin and strengthening CTNNB1/USF1 binding to the miR-483 promoter, resulting in increased miR-483-3p expression and repression of the pro-apoptotic gene *BBC3*/*PUMA*, thereby contributing to tumor progression and chemoresistance, particularly in liver cancer [47]. Early growth response protein 1 (EGR1) has been shown to upregulate miR-483 expression via a ceRNA mechanism involving the *HMGA1P7* pseudogene [48]. Overexpression of *HMGA1P7* activates EGR1, which in turn enhances miR-483 levels—a process associated with tumor progression and poor prognosis in several human carcinomas [48]. Moreover, KLF9 exerts independent transcriptional control over miR-483-3p by binding directly to upstream regulatory elements of the miR-483 locus, a process distinct from *IGF2* transcription [49]. This mechanism was validated via ChIP-seq analysis in testicular seminoma cells, where the downregulation of KLF9 resulted in repressed miR-483-3p expression, thereby impairing its ability to inhibit cancer cell proliferation and invasion [49].

Except for independent regulation, miR-483 can also be regulated synergistically with its host gene *IGF2*, where the tissue-specific transcription factor ZBED6 exerts a suppressive role by binding to a conserved GCTCGC motif within intron 1 of *IGF2* [50]. Disruption of ZBED6 binding releases transcriptional repression of both *IGF2* and the intronic miR-483 locus, leading to coordinated upregulation of miR-483-3p/5p in skeletal muscle and kidney tissues [50]. Similarly, WT1 typically suppresses *IGF2* transcription; thus, WT1 mutations or functional loss led to increased expression of both *IGF2* and miR-483, prominently observed in Wilms tumors [42]. For transcriptional activation, KLF4 exerts co-regulatory control over both *IGF2* and miR-483 through methylation-dependent binding to the *IGF2*-DMR0 region, thereby coordinating their expression via shared epigenetic mechanisms [78]. This dual regulation significantly impacts downstream pathways, including connective tissue growth factor (CTGF) signaling, ultimately modulating disease progression by suppressing epithelial–mesenchymal transition (EMT) [34]. Beyond transcriptional regulation, miR-483 also contributes to tumor epigenetic deregulation, including disruption of imprinting at the *IGF2* locus through altered chromatin modification and loss of CTCF and SUZ12 binding [79]. These findings highlight a multilayered regulatory axis in which transcription factors control miR-483 expression through sequence-specific promoter targeting and broader epigenetic modifications at the locus. Current research on TF-mediated regulation of miR-483 concentrated mainly on oncological contexts. Mechanistic studies in cardiovascular and metabolic diseases are still lacking and warrant further investigation.

### 2.2. CircRNA-miR-483 Regulation

CircRNAs can be molecular sponges that bind and sequester specific miRNAs from their target mRNAs [81]. This form of post-transcriptional regulation has gained considerable attention in the modulation of miRNA activity across diverse pathological conditions. Evidence suggests several circRNAs regulate miR-483 isoforms, influencing downstream gene expression and disease processes in cardiovascular disorders, autoimmune diseases, fibrosis, neuropathic pain, and bone metabolism.

Specific circRNAs have been found to modulate miR-483 activity in cardiovascular pathologies with pathogenic consequences. For example, circ_0000006 is significantly upregulated in aortic dissection tissues and functions as a sponge for miR-483-5p, thereby reducing its inhibitory effect on the target gene *KDM2B*. This reduction in miR-483-5p-mediated inhibition of *KDM2B* promotes the proliferation and phenotypic switching of vascular smooth muscle cells, contributing to the progression of aortic dissection [51]. Similarly, circ_0122153 was upregulated in patients with essential hypertension and shows a negative correlation with miR-483-3p expression. By sponging miR-483-3p, circ_0122153 disrupts the renin–angiotensin–aldosterone system (RAAS), leading to aldosterone dysregulation and elevated blood pressure [52].

In autoimmune disorders, circ_0123190 is downregulated in lupus nephritis tissues and negatively correlates with miR-483-3p. Its reduced expression leads to increased activity of miR-483-3p, which excessively represses *APLNR*, thereby exacerbating renal inflammation and fibrosis [46].

Circular RNAs have also been found to regulate miR-483 activity in both fibrotic and metabolic bone diseases [53,54,55,57]. In cirrhotic cardiomyopathy (CCM), circ-ASAP1 is significantly downregulated following liver transplantation, resulting in the suppression of miR-483-3p. The consequent increase in miR-483-3p activity contributes to cardiac dysfunction by modulating the mTOR/MAPK signaling pathways [53]. In osteoporosis, circ_0006859 inhibits osteogenic differentiation of bone marrow mesenchymal stem cells (BMSCs) by targeting miR-483-3p [54]. This circRNA downregulates miR-483-3p, leading to upregulation of *EFNA2* and *DOCK3*, impairing osteogenic differentiation of BMSCs via the Wnt signaling pathway. This mechanism contributes to the progression of osteoporosis by impairing osteoblast differentiation and promoting bone loss in postmenopausal osteoporosis [54].

CircUTRN24 has been implicated in regulating biliary atresia through the circUTRN24/miR-483-3p/*IGF1* axis in the context of liver fibrosis [55]. This axis modulates autophagy-related pathways and influences fibrosis progression by activating the mTOR signaling pathway, which plays a crucial role in hepatic stellate cell (HSC) autophagy and fibrotic changes [55]. Additionally, in chronic inflammatory visceral pain, moxibustion therapy has been shown to upregulate circRNA_02767 in the spinal cord, which sponges miR-483-3p to increase *GFAP* expression [57]. This interaction alleviates central sensitization and reduces neuropathic pain, highlighting a novel miR-483-mediated analgesic mechanism [57].

Together, these findings illustrate a growing recognition of circRNAs as modulators of miR-483 function in human diseases. Through ceRNA-mediated sequestration, circRNAs influence the biological activity and downstream effects of miR-483, adding an epigenetic regulatory layer with both mechanistic and therapeutic implications. This highlights the potential of circRNA–miR-483 networks as diagnostic biomarkers and targets for intervention across diverse pathological states.

### 2.3. LncRNA-miR-483 Regulation

Long non-coding RNAs are critical upstream regulators of miR-483 through ceRNA mechanisms, modulating miR-483 function across diverse pathophysiological contexts, including cancers, metabolic diseases, and cardiovascular disorders [58,59,60,61,62,63]. A common regulatory mechanism involves lncRNA-mediated miRNA sponging, effectively sequestering miR-483 isoforms and releasing their downstream target genes from suppression [81]. For instance, in osteosarcoma, lncRNA *NEAT1* competitively binds miR-483, relieving miR-483-mediated inhibition of *STAT3*, thereby promoting epithelial–mesenchymal transition (EMT) and metastasis [58]. Similarly, *NEAT1* has been associated with prostate cancer progression through a ceRNA network involving miR-483-3p and *UBE2C* [59]. *NR2F1-AS1* also exerts oncogenic effects by sponging miR-483-3p in acute myeloid leukemia (AML), enhancing *IGF1* expression and fostering azacitidine resistance [60]; in osteosarcoma, *NR2F1-AS1* targets miR-483-3p to upregulate *FOXA1*, driving malignant progression [61].

Additional cancer-related interactions include glioma, where *LINC00662* promotes tumor proliferation and invasiveness by sequestering miR-483-3p, increasing *SOX3* expression [62]. In triple-negative breast cancer, *MIR4500HG003* sponges miR-483-3p to enhance MMP9-driven metastasis [63]. *SNHG11* facilitates gastric cancer progression by binding miR-483-3p, thus activating Wnt/β-catenin and *ATG12*-mediated autophagy pathways [64]. *BCAR4* enhances colorectal cancer chemotherapy resistance by releasing *RAB5C* from miR-483-3p suppression [65], while *TTC39A-AS1* promotes breast cancer cell proliferation and metastasis via miR-483-3p-dependent regulation of *MTA2* [45]. In contrast, *LINC00908* inhibits prostate cancer cell proliferation and metastasis by competitively binding miR-483-5p, thus elevating *TSPYL5* expression [66]. *MEG3* interacts with miR-483-3p in hepatocellular carcinoma under high-glucose conditions, promoting *ERp29* expression and cellular proliferation and migration [67]. Additionally, *H19* interacts with miR-483-5p, upregulating *DUSP5* expression to mitigate mechanical stress-induced cartilage degradation [68], and separately promotes osteogenic differentiation in periodontal ligament stem cells by targeting miR-483-3p to activate Wnt/β-catenin signaling [69].

Beyond cancer, lncRNA-miR-483 interactions are significant in metabolic and cardiovascular disorders [70,71,72,73]. *SNHG14* exacerbates diabetic kidney disease by sponging miR-483-5p, thus derepressing *HDAC4* and promoting renal tubular damage, inflammation, and fibrosis [70]. *MALAT1* negatively regulates miR-483-3p in acute cerebral infarction, enhancing inflammation via hs-CRP [71]. Additionally, in type 2 diabetes mellitus complicated by coronary artery disease, *DBH-AS1* affects coronary artery endothelial cell function by modulating miR-483-5p activity, influencing cell proliferation, apoptosis, and inflammatory cytokine secretion [72]. *SNHG29* drives chronic myeloid leukemia progression through miR-483-3p targeting, activating the PI3K/Akt signaling pathway via upregulated *CBL* [73].

A unique mechanism distinct from typical sponging involves the lncRNA *MPRL* (NR_034085), which directly binds cytoplasmic pre-miR-483-5p, inhibiting its processing and modulating mitochondrial dynamics to enhance cisplatin sensitivity in tongue squamous cell carcinoma [74]. Moreover, adding complexity to the regulatory landscape, miR-483-5p itself can act upstream in HCC by binding the *IGF2*/*H19* enhancer, forming chromatin loops via MED1, and thereby transcriptionally upregulating both its host gene *IGF2* and the neighboring oncogenic lncRNA *H19*, promoting malignancy [75]. Overall, these extensive interactions highlight the broad functional versatility and clinical significance of lncRNA-miR-483 interactive networks across diverse human diseases, warranting further research to elucidate their therapeutic potential.

## 3. Integrative miR-483-Mediated Networks

### 3.1. Systematic Regulatory Network Mediated by miR-483

To thoroughly elucidate the regulatory role of miR-483 across various human diseases, we systematically collected and integrated experimentally validated interactions involving miR-483 from 146 published studies in PubMed from 2009 to 2025. Our search strategy utilized keywords such as “microRNA-483”, “miR-483”, in combination with terms like “target”, “regulation”, “long non-coding RNA”, “lncRNA”, “circular RNA”, “circRNA”, “transcription factor” and “TF”. Papers were selected with several criteria: (1) involved miR-483 expression profiling studies, (2) used experimental methods, and (3) reported miR-483-target relationships. Thus, studies that are purely bioinformatics and lack control experiments, as well as review articles, were excluded.

While compiling these data, a central challenge emerged: the expression profile of miR-483 is profoundly heterogeneous across the literature. This widespread heterogeneity is strongly corroborated by our pan-cancer analysis of The Cancer Genome Atlas (TCGA), which reveals substantial expression variability not only between different cancer types but also, critically, among individual patients within the same malignancy (Supplementary Figure S1). This high degree of patient-to-patient variability, likely due to factors such as cellular heterogeneity or technical differences, means that relying on average expression trends can be misleading [82]. Therefore, we concluded that a more robust understanding of miR-483 must be built upon its conserved, experimentally validated functional impacts. By concentrating on these core functions and targets, we can identify the fundamental principles of its action that transcend the “noise” of context-specific expression. A detailed summary of the reported dysregulation of miR-483 in various diseases [62,83,84,85,86,87,88,89,90,91,92,93,94,95,96,97,98,99,100,101,102,103,104,105,106,107,108,109,110,111,112,113], is provided in Supplementary Table S1, summarizing the miR-483’s expression patterns, target genes, cell lines, and functions in specific disease conditions.

**Figure 3:**
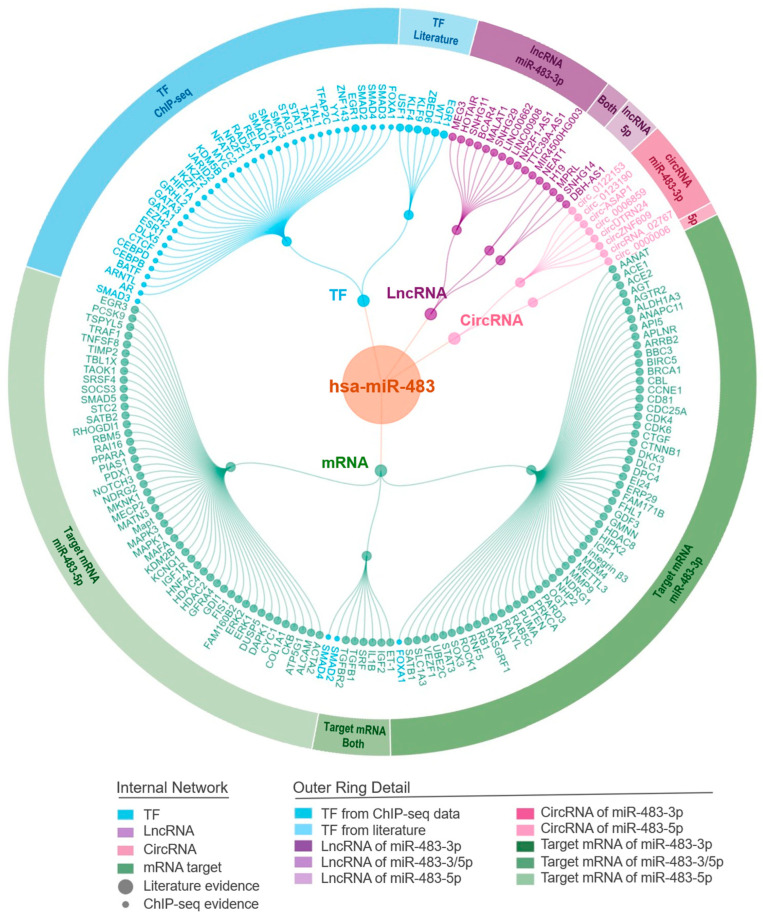
Integrative regulatory network centered on miR-483. The network integrates 65 upstream regulators (circRNAs, lncRNAs, and TFs) and 114 downstream targets (58 specific to miR-483-3p, 48 to miR-483-5p, 8 shared). Node colors of internal network indicate regulatory roles: blue for transcription factors, purple for lncRNAs, pink for circRNAs, green for mRNA targets. Size of nodes indicated the evidence level of interactions. Outer ring provides detailed classifications for subset groups. Network visualization performed using R software (v4.5.2).

The integrative regulatory network presented in Figure 3 comprises 42 validated TF-miR-483 interactions, of which 36 additional interactions were supported by ChIP-seq assays and obtained by cross-referencing data from the miRStart2 [114] and TransmiR [115] databases. Additionally, we identified 7 circRNA-miR-483 interactions and 16 lncRNA-miR-483 interactions directly from the literature. Downstream, we incorporated 114 experimentally validated mRNA targets collected from published literature, comprising 58 unique targets for miR-483-3p, 48 unique targets for miR-483-5p, and 8 targets shared by both isoforms. All interactions were rigorously validated according to the standards set by miRTarBase, relying only on literature based robust experimental evidence such as luciferase reporter assays, quantitative PCR (qPCR), or Western blot.

Further analysis of the downstream regulatory network highlights that miR-483 isoforms (miR-483-3p and miR-483-5p) exert distinct biological functions via their specific mRNA targets. miR-483-3p targets prominently include genes such as *AGT*, *ACE1*, *ACE2*, *AGTR2*, and *MMP9*, whereas miR-483-5p notably targets genes like *ALCAM*, *ATP5G1*, *CKB*, and *SRF*. Notably, FOXA1, SMAD2, and SMAD4 exhibit bidirectional regulatory relationships with miR-483, functioning as transcription factors that regulate miR-483 expression while simultaneously serving as downstream targets: FOXA1 for miR-483-3p alone, and SMAD2/SMAD4 for both isoforms. This reciprocal regulation creates potential feedback loops that may amplify or attenuate miR-483-mediated signaling. These targets collectively underline the crucial involvement of miR-483 in diverse physiological and pathological pathways, including cardiovascular regulation, tumorigenesis, cell metabolism, and metastasis. The detailed and structured overview presented in the network (Figure 3) provides robust evidence and a clear regulatory framework, laying a solid foundation for subsequent analyses.

### 3.2. Disease-Specific Pathway Signatures of the miR-483 Network

**Figure 4:**
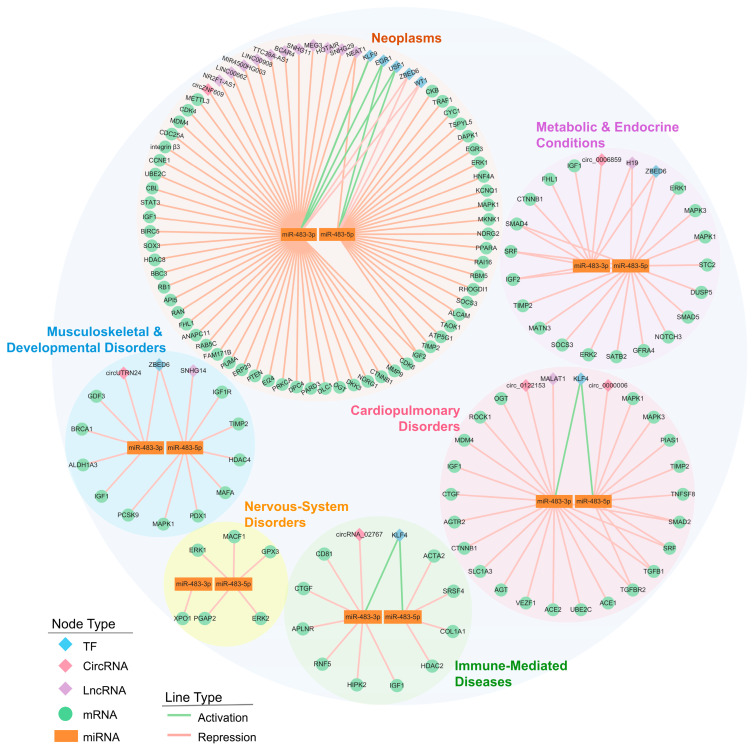
miR-483 regulated six disease-specific subnetworks: neoplasms, cardiopulmonary, metabolic & endocrine, immune/autoimmune, nervous system, and musculoskeletal & developmental. Node shapes and colors represent regulatory categories: blue squares for transcription factors, pink squares for circRNAs, purple squares for lncRNAs, green circles for mRNA targets, and yellow rectangles for miRNAs. Edge colors indicate the mode of regulation: green edges represent transcriptional activation of miR-483 by upstream TFs, while red edges denote repressive interactions, including lncRNA/circRNA sequestration or miRNA-mediated mRNA suppression.

To pinpoint disease-relevant control modules within the global miR-483 network, we partitioned all experimentally supported interactions into six disease-specific subnetworks: neoplasms, cardiopulmonary disorders, metabolic and endocrine conditions, immune-mediated diseases, nervous-system disorders, and musculoskeletal and developmental disorders (Figure 4).

#### 3.2.1. Neoplasms

In cancer, miR-483 acts as an oncogene by suppressing tumor-suppressive checkpoints across three functional axes: (i) cell-cycle acceleration coupled with apoptosis evasion; (ii) induction of EMT through Wnt/β-catenin and TGF-β/SMAD pathways; and (iii) amplification of growth-factor signaling and extracellular matrix remodeling that drive invasion and metastasis. Together, these programs establish a progression sequence: proliferation precedes phenotypic plasticity, which in turn enables dissemination.

(i) Cell-Cycle Acceleration and Apoptosis Evasion. Functionally, miR-483-3p disrupts the cellular division checkpoint and apoptotic brakes while reinforcing cyclin/CDK drive: it targets multiple cell-cycle regulators, including *CCNE1*, *CDK4*/*6*, *CDC25A*, and *RB1*, thereby accelerating G1/S transition [37,56,116,117,118]. Simultaneously, it suppresses the pro-apoptotic factor *BBC3*/*PUMA* and the p53 regulator *MDM4*, shifting the balance toward proliferation and apoptosis resistance [42,119,120]. Consistent with this target profile, overexpression of miR-483-3p inhibits TP53-mediated apoptosis in hepatocellular carcinoma [121]. This dual repression, of both division checkpoints and death signals, permits sustained tumor expansion.
(ii) Developmental Plasticity and EMT via Wnt/β-Catenin and TGF-β/SMAD Pathways. miR-483-3p stabilizes Wnt signaling by targeting the pathway inhibitor *DKK3*, a mechanism implicated in colorectal and gastric cancer progression [64,122]. In parallel, it represses *SMAD4* and *SMAD2*, central transducers of TGF-β signaling [123,124]. The functional outcome of SMAD suppression depends on tumor context: in early-stage cancers where TGF-β retains growth-inhibitory activity, miR-483 relieves this brake; in advanced tumors, reduced canonical SMAD signaling may favor non-canonical, pro-invasive TGF-β outputs that promote EMT.
(iii) Growth-Factor Signaling and Invasion-Metastasis Circuits. Both miR-483 isoforms enhance mitogenic signaling by targeting negative regulators such as *PTEN* and by directly modulating effectors like *IGF1* and *MAPK1*/*ERK2*, thereby reinforcing PI3K-Akt and MAPK cascades [29,73,125,126,127]. Downstream effects include increased *eIF4E*-mediated protein synthesis, which supports rapid cell growth [127]. Invasion is further promoted through extracellular matrix remodeling: miR-483-3p targets *MMP9* and the integrin *ITGB3*, facilitating basement membrane degradation and cell motility [49,63,128]. Additional effects on genome maintenance and chromatin programs (e.g., BRCA1, histone deacetylases) may confer resistance to genotoxic stress [16].

This three-axis model describes a temporal progression: miR-483 first disables proliferative restraints, then induces phenotypic plasticity through developmental signaling pathways, and finally enables invasion via growth-factor amplification and matrix degradation.

While the oncogenic axes described above represent the core functional logic of miR-483 in cancer, the specific molecular players can differ between tumor types. To dissect these context-specific roles further, we constructed regulatory sub-networks for 15 malignancies by analyzing TCGA data [115,129,130,131,132,133,134,135,136]. These networks, detailed in Supplementary Figure S2, reveal a profound heterogeneity in the miR-483 interactome across different cancer types. This diversity is evident across multiple regulatory layers. First, the upstream regulatory landscape shifts dramatically between tissues: for instance, the Bladder Cancer (BLCA) network is driven by the dysregulation of transcription factors such as MYOD1, whereas the Liver Cancer (LIHC) network displays a distinct regulatory signature involving ESR1. Second, the availability of downstream targets determines the effector output. While CKB emerges as a convergent target downregulated by both miR-483-3p and -5p in Stomach Cancer (STAD), it is notably absent from the Pheochromocytoma and Paraganglioma (PCPG) network, which instead features the significant dysregulation of neural-lineage genes like SOX3. Finally, the topological complexity itself varies: the networks range from the dense, multi-layered interactome observed in LIHC to the sparser networks in PCPG. Together, these patterns outline how miR-483 deploys different regulatory repertoires in different tumor environments, providing a mechanistic basis for its context-dependent oncogenic behavior.

#### 3.2.2. Cardiopulmonary Disorders

Unlike its oncogenic role, miR-483 exhibits predominantly protective functions in cardiopulmonary diseases through three mechanisms: (i) modulation of the renin–angiotensin–aldosterone system (RAAS) to regulate blood pressure and vascular tone; (ii) suppression of TGF-β/SMAD-ROCK signaling to limit cardiac and vascular fibrosis; and (iii) enhancement of cardiomyocyte survival and angiogenic balance under ischemic stress.

(i) RAAS Modulation and Vascular Homeostasis. The RAAS pathway, which controls blood pressure and fluid balance, is uniquely targeted by miR-483 in cardiovascular contexts [137]. miR-483-3p directly represses *AGT* (angiotensinogen), *ACE* and *ACE2* (angiotensin-converting enzymes), and *AGTR2* (angiotensin II receptor type 2), thereby attenuating hypertensive signaling and pathological cardiac remodeling [138]. miR-483-5p complements this activity by targeting *MAPK1*/*3* (*ERK2*/*ERK1*) and the vasoconstrictor endothelin-1 (*ET-1*), which interface with RAAS to regulate smooth muscle contractility and endothelial function [139,140]. Together, both isoforms coordinate to dampen pressor signaling and preserve vascular stability.
(ii) Antifibrotic Control via TGF-β/SMAD-ROCK Signaling. Cardiac fibrosis results from sustained TGF-β signaling and cytoskeletal remodeling in myofibroblasts. miR-483-5p suppresses this cascade at multiple nodes: it targets the ligand *TGFB1*, the receptor *TGFBR2*, and the downstream effector *SMAD2*, thereby reducing profibrotic gene transcription [140,141]. Concurrently, miR-483-5p inhibits ROCK1, a kinase that drives actomyosin contractility and myofibroblast differentiation, which limits extracellular matrix deposition and tissue stiffening [140]. Consistent with this mechanism, miR-483-5p also downregulates *TIMP2* and *PDGFB*, matrix regulators implicated in cardiac remodeling [140,142]. This multilayered repression establishes miR-483-5p as a central antifibrotic regulator in the heart.
(iii) Stress-Apoptosis Control and Angiogenic Balance. miR-483-5p enhances cardiomyocyte survival under stress by targeting *MAPK3*/*ERK1*, which modulates cytoprotective signaling, and by suppressing the pro-apoptotic factor *TNFSF8* [143]. In the vasculature, miR-483-3p regulates the endothelial transcription factor VEZF1, promoting orderly angiogenesis and barrier integrity [144]. These effects sustain both myocardial viability and microvascular function during ischemic injury.

In summary, miR-483 stabilizes vascular tone through RAAS inhibition, limits pathological remodeling via TGF-β suppression, and protects cardiac tissue from stress-induced injury, collectively maintaining cardiopulmonary homeostasis. Emerging evidence further suggests that the protective functions of miR-483 are themselves regulated by redox state: oxidative stress induces 8-oxo-guanosine modification of miR-483, altering its seed sequence and redirecting it from vasculoprotective targets (*CTGF*, *PCSK9*) toward pro-atherogenic targets (*KLF4*) [28]. This epitranscriptomic switch couples miR-483 activity to cellular oxidative status, adding an additional layer of context-dependency in cardiovascular disease.

#### 3.2.3. Metabolic & Endocrine Conditions

In metabolic and endocrine disorders, miR-483 regulates three interconnected processes: (i) β-cell function and insulin/IGF signaling, which are central to glucose homeostasis; (ii) inflammatory JAK/STAT pathways that drive diabetic complications; and (iii) adipogenesis and lipid metabolism, largely through PPAR-dependent mechanisms.

(i) β-Cell Identity and Insulin/IGF Signaling. miR-483-5p targets *PDX1* and *MAFA*, transcription factors required for β-cell maturation and insulin gene expression [145]. Additionally, miR-483-3p represses *IGF1* and *IGF1R*, while miR-483-5p targets the downstream kinase *MAPK1*/*ERK2*, collectively modulating β-cell survival and glucose-stimulated insulin secretion in experimental diabetes [36,146,147].
(ii) Inflammatory JAK/STAT Signaling and Diabetic Complications. By tuning cytokine signaling nodes such as SOCS3 and IL6, miR-483-5p links metabolic inflammation to endocrine dysfunction [148,149]. This axis extends to tissue injury, in diabetic kidney disease, miR-483-5p suppresses *TIMP2* and *HDAC4*, attenuating TGF-β-driven renal fibrosis [36,146,147]. Repression of *IGF1R* further connects metabolic inflammation to diabetic retinopathy, suggesting that miR-483 coordinates immune-metabolic crosstalk across multiple target organs.
(iii) Adipogenesis and Lipid Metabolism. miR-483-5p influences adipocyte differentiation by targeting *ALDH1A3* and regulates cholesterol metabolism through *PCSK9* [150,151,152]. These targets converge on pathways controlled by PPAR transcription factors [153], providing a mechanistic basis for miR-483 effects on adipose expansion, circulating lipid profiles, and hepatic lipid deposition, contributing to obesity, NAFLD, and cardiometabolic risk.

Collectively, miR-483 integrates β-cell function, inflammatory signaling, and lipid metabolism, positioning it as a regulator of glucose homeostasis and diabetic end-organ damage.

#### 3.2.4. Immune-Mediated Diseases

In immune-mediated diseases such as systemic sclerosis and lupus nephritis, miR-483 participates in the regulation of fibrosis and inflammation through complex and context-dependent mechanisms: (i) modulation of TGF-β/SMAD signaling and extracellular matrix (ECM) regulators; and (ii) influence on cytokine-related networks and leukocyte dynamics.

(i) Modulation of the Pro-Fibrotic Cascade. In acute or immune-related fibrosis with transient inflammation such as pancreatitis-associated lung injury, rheumatoid arthritis, and sepsis-induced intestinal injury, miR-483 acts as a suppressor of pathological fibrosis and inflammation [154,155,156]. Both miR-483 isoforms interfere with TGF-β–driven signaling cascades: miR-483-3p represses upstream amplifiers of fibrotic signaling such as *CTGF* and the nuclear kinase *HIPK2*, while miR-483-5p sustains TGF-β-driven transcription by suppressing the splicing regulator *SRSF4* and epigenetic cofactor *HDAC2*, thereby blocking myofibroblast differentiation and reducing the expression of structural proteins such as COL1A1 [34,154,155,156,157]. These effects collectively limit ECM accumulation and tissue stiffening, aligning miR-483 with an anti-fibrotic and anti-inflammatory role in acute or immune-driven injury.
  By contrast, in chronic fibrotic disorders such as systemic sclerosis (SSc), miR-483-5p appears to engage a different regulatory axis [158]. Overexpression of miR-483-5p in endothelial cells enhances transcription of myofibroblast differentiation markers αSMA and SM22A, while suppresses *FLI1*, a negative regulator of ECM that is downregulated in SSc skin, indicating a selective remodeling rather than a global repression of ECM synthesis [158]. Together, these data position miR-483-5p as a context-dependent regulator of fibrogenesis, displaying protective effects in acute immune-inflammatory injury but pro-fibrotic remodeling in chronic sclerotic conditions.

(ii) Cytokine Signaling and Leukocyte Dynamics. miR-483 also modulates the inflammatory processes that initiate and sustain fibrosis [46]. miR-483-3p targets *CD81* (involved in immune cell adhesion and signaling), *RNF5* (a regulator of inflammatory signaling), and *APLNR* (which controls leukocyte extravasation from blood into tissue) [46,159]. Additionally, repression of *IGF1* influences fibroblast-macrophage crosstalk, a critical determinant of tissue repair and inflammation [160]. Through these targets, miR-483-3p attenuates both the intensity of cytokine signaling and the extent of leukocyte infiltration, thereby aligning the inflammatory state with the tissue remodeling processes.

Together, these mechanisms position miR-483 as a homeostatic regulator of fibrogenesis, fine-tuning ECM composition and influencing fibroblast-endothelial interactions depending on the cellular environment and disease stage.

#### 3.2.5. Nervous-System Disorders

In nervous-system disorders, miR-483 functions as a fundamentally neuroprotective molecule. Its targets converge on two molecular programs: (i) attenuation of chronic neurodegeneration, exemplified by regulation of Tau pathology in Alzheimer’s disease; and (ii) acute cytoprotection against oxidative and excitotoxic stress. The dual functions position miR-483 as a regulator of both neuronal survival and synaptic integrity.

(i) Regulating Tau Phosphorylation and Synaptic Plasticity via MAPK/ERK Signaling. In Alzheimer’s disease models, miR-483-5p targets *ERK1* and *ERK2*, kinases that drive pathological Tau hyperphosphorylation [161]. Notably, this repression occurs within a range that limits toxic Tau phosphorylation without abolishing ERK-dependent synaptic plasticity, a balance critical for preserving long-term potentiation and cognitive function. Thus, miR-483-5p may uncouple neurodegenerative ERK signaling from physiological synaptic maintenance.
(ii) Stress-Apoptosis Buffering and Neuronal Survival. miR-483 confers acute stress resistance through coordinated regulation of oxidative defense and apoptotic checkpoints [162,163]. miR-483-5p limits oxidative damage by targeting *GPX3* and modulates excitotoxicity via MAPK/ERK fine-tuning, while miR-483-3p suppresses *XPO1* to retain pro-survival transcription factors in the nucleus [162,163]. Over longer timescales, both isoforms sustain synaptic architecture: miR-483-3p and -5p regulate *XPO1* and *PGAP2*, genes required for neurotrophic signaling and synaptosomal protein trafficking [163]. This two-tiered mechanism: immediate cytoprotection plus sustained structural support, distinguishes miR-483 from stress-response miRNAs with purely acute effects.

Collectively, these data suggest miR-483 mimics warrant evaluation in preclinical models of stroke, traumatic brain injury, and Alzheimer’s disease, though delivery to the central nervous system remains a technical barrier.

#### 3.2.6. Musculoskeletal & Developmental Disorders

In musculoskeletal development and regeneration, miR-483 regulates cell fate decisions through two major programs: (i) skeletal patterning and lineage commitment, where the two isoforms exert opposing effects on osteogenesis, and (ii) myogenesis and muscle repair, controlled via IGF signaling and myogenic transcription factors.

(i) Regulation of Skeletal Patterning and Lineage Commitment. The two miR-483 isoforms have opposite roles in bone formation. miR-483-5p inhibits osteogenesis by repressing SATB2, a chromatin regulator that activates the RUNX2/osteocalcin transcriptional program required for osteoblast differentiation [164]. It also suppresses osteogenic differentiation of bone marrow mesenchymal stem cells (BMSCs) by targeting *RPL31*, which modulates RAS/MEK/ERK signaling [38].
  Conversely, miR-483-3p promotes bone formation by repressing *DKK2*, an inhibitor of Wnt signaling, thereby enhancing osteoblast proliferation and differentiation [165]. miR-483-3p also facilitates BMSC osteogenesis by targeting STAT1 [166].
  Beyond osteogenesis, miR-483 regulates chondrogenesis and skeletal morphogenesis. miR-483-3p inhibits chondrogenic differentiation by targeting *SMAD4* [167], while miR-483-5p modulates cartilage homeostasis by repressing *MATN3*, a cartilage matrix protein [168], and repressed *DUSP5*, a MAPK phosphatase that provides spatial patterning signals during limb and joint formation [169].
(ii) Myogenesis and Tissue Regeneration. In skeletal muscle, miR-483 attenuates anabolic IGF signaling by targeting *IGF1*, *IGF2*, and their downstream kinases *MAPK1*/*ERK2* and *MAPK3*/*ERK1*, thereby reducing signals for myocyte survival, hypertrophy, and protein synthesis [32,169,170]. Both isoforms also repress the serum response factor (SRF), a transcription factor controlling actin cytoskeleton and contractile gene expression, and NOTCH3, which targeted by miR-483-5p, regulating satellite cell activation during muscle repair [148,171]. This regulatory network is further refined through the targeting of *DUSP5* by miR-483-5p, which controls ERK signal duration and balances proliferation with terminal differentiation [68].

Through these targets, miR-483 coordinates skeletal patterning, cartilage development, and muscle regeneration across craniofacial, axial, and appendicular structures.

#### 3.2.7. The Functional Landscape of miR-483 in Diverse Human Diseases

**Figure 5:**
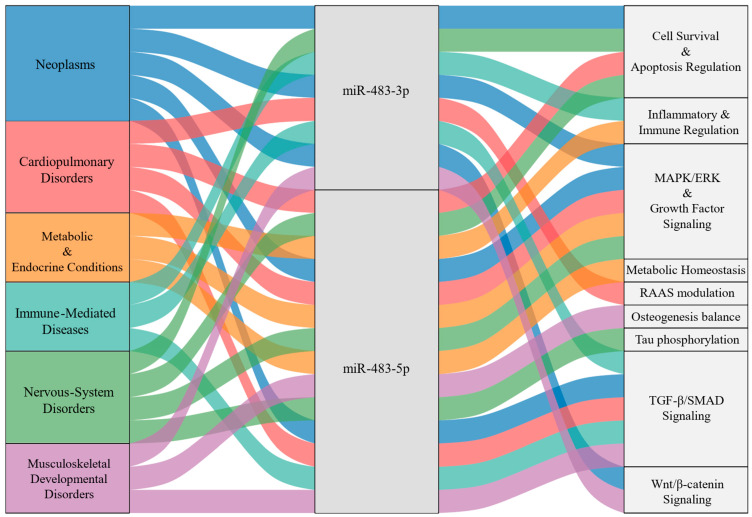
Functional landscape of miR-483 downstream regulation across human diseases. The Sankey diagram illustrates the regulatory connections between six major disease categories (left column), the two arms of miR-483 (center column), and their downstream biological pathways (right column). The flows represent the functional linkage from a disease context, through a specific miRNA arm, to a regulated pathway. The color of each flow is determined by the originating disease category, allowing for the visual tracing of context-specific functions throughout the network.

As summarized in Figure 5, miR-483 follows a “shared backbone with context-specific branches.” Across diseases, it repeatedly engages three core pathways: MAPK/ERK with growth-factor signaling [73,125], TGF-β/SMAD signaling [140], and cell survival-apoptosis balance [119]. By fine-tuning these common hubs, miR-483 produces distinct outcomes in different tissues, it promotes proliferation in neoplasms while supporting cell survival and stability in cardiopulmonary and nervous-system disorders. This illustrates how a single miRNA can elicit context-appropriate effects by adjusting the same underlying circuitry.

At the same time, miR-483 displays context specificity. In cardiopulmonary disease, it links to RAAS modulation, a pathway tightly tied to blood pressure and vascular control [138]; in nervous-system disorders, it influences Tau phosphorylation and synaptic plasticity [161]; and in metabolic and musculoskeletal conditions, it connects to metabolic homeostasis/insulin signaling and osteogenesis balance, respectively [68,145]. This strategy explains how miR-483 can govern shared cellular processes yet precisely tailor tissue-specific functions that drive disease phenotypes. A summarization of key validated targets of miR-483 across these six disease categories is provided in Table 2.

## 4. miR-483 as a Clinical Biomarker and Therapeutic Target

The consistent dysregulation of miR-483 across numerous pathologies has positioned it as a promising clinical biomarker and therapeutic target. Its levels in both tissue and circulation correlate with disease presence, progression, and patient outcomes. Consequently, strategies to modulate miR-483 activity through inhibition or restoration are emerging as a compelling therapeutic avenue.

### 4.1. Diagnostic Applications

Circulating miR-483 levels, particularly those of miR-483-5p, have shown considerable promise as noninvasive diagnostic biomarkers [30,31,32,33]. In oncology, its utility is most pronounced in distinguishing malignant from benign or healthy tissue [172,173,174]. For instance, elevated plasma miR-483-5p can help identify patients with early-stage gastric cancer, and even precancerous lesions, from those with gastritis, offering a potential blood-based screening tool to complement endoscopy [172]. In adrenocortical carcinoma (ACC), significantly upregulated serum miR-483-5p is a reliable marker for differentiating malignant ACC from benign adrenal adenomas preoperatively [173]. A comparative study also found plasma miR-483-5p was overexpressed in ACC vs. adenomas [174]. Thus, measuring circulating miR-483-5p aids in diagnosing of adrenal cancer, complementing imaging and hormone tests.

Elevated miR-483-5p has diagnostic relevance beyond cancer. In multiple myeloma (MM) patients, plasma miR-483-5p is higher than in healthy individuals, with an ROC AUC of ~0.745 for distinguishing MM [175]. While altered miR-483 expression has also been noted in metabolic conditions like type 2 diabetes, its diagnostic utility in non-cancerous diseases remains under investigation. Overall, accumulating evidence (from profiling studies and validation in independent cohort) supports miR-483-5p as a circulating biomarker for diverse pathologies, especially cancers.

### 4.2. Prognostic Significance

Beyond early diagnosis, miR-483 expression levels provide critical prognostic information, often correlated with disease aggressiveness and patient survival [176,177]. In nasopharyngeal carcinoma (NPC), high tumor expression of miR-483-5p is a powerful independent predictor of poor 5-year overall (~55% vs. 87% in the low miR-483 group) and progression-free survival [177]. This clinical observation is mechanistically supported by findings that miR-483-5p suppresses the tumor suppressor EGR3 to promote NPC cell invasion [177]. In ACC, post-surgery miR-483-5p levels predict tumor recurrence. One study of ACC patients found that those who relapsed within 3 years had ~4-fold higher miR-483-5p in serum at 3 months postoperatively than those without recurrence. A threshold of ~7.5 × 10^5^ copies/mL correctly stratified high-risk patients with 100% specificity (and ~62% sensitivity). This makes circulating miR-483-5p a potent prognostic biomarker for ACC, outperforming some conventional measures [173].

The association between elevated miR-483 and poorer outcomes is a recurring theme across multiple cancer types, including pediatric Wilms’ tumor (miR-483-3p) and multiple myeloma (miR-483-5p) [29,126,175]. These findings suggest that monitoring miR-483 levels could be a valuable tool for stratifying patient risk and predicting disease course. Our pan-cancer survival analysis using TCGA data lends further support to this idea, while also revealing a crucial layer of complexity. We found that high miR-483 expression predicts unfavorable survival in cancers like bladder and esophageal carcinoma, yet is associated with favorable outcomes in kidney and liver cancer (Supplementary Figure S3).

This observation in patient survival can be explained by the underlying molecular networks active in these tumors (Supplementary Figure S2). For instance, in bladder cancer, where high miR-483 predicts poor survival, our analysis reveals an upregulated, oncogenic sub-network with miR-483 targeting genes involved in proliferation and cell cycle. Conversely, in kidney clear cell carcinoma, where higher miR-483 is protective, it is part of a downregulated, tumor-suppressive network, suggesting its loss contributes to disease progression. This integration of survival and network data reinforces the principle of context-dependent functionality, highlighting that the regulatory landscape of a specific cancer dictates the prognostic role of miR-483.

### 4.3. Therapeutic Potential

Given its functional role in driving malignancy, modulating miR-483 activity is a rational and promising therapeutic strategy. The approach is twofold: inhibiting oncogenic miR-483 arms with antagomiRs or restoring tumor-suppressive arms with synthetic mimics.

Restoration therapy appears promising for overcoming drug resistance [178]. In EGFR-mutant non-small cell lung cancer (NSCLC), resistance to tyrosine kinase inhibitors (TKIs) is associated with the epigenetic silencing of the tumor-suppressive miR-483-3p [179]. Preclinical studies show that reintroducing miR-483-3p with mimics can re-sensitize resistant cells to gefitinib by reversing EMT and inhibiting cell migration [179]. This positions miR-483-3p mimics as a potential combination therapy to overcome acquired TKI resistance in lung cancer [179].

Conversely, inhibition therapy has been explored for cancers where miR-483 is oncogenic. In aggressive tumors like NPC and ACC, where high miR-483-5p drives proliferation and metastasis, knockdown with anti-miR-483-5p inhibitors (siRNA or antagomir) in preclinical models has been shown to slow tumor growth, induce apoptosis, and impair metastatic behavior [180]. While these approaches are not yet in clinical trials, proof-of-concept has been established in xenograft models, and research into effective delivery systems, such as nanoparticles or exosomes, is underway.

However, the path to clinical translation is fraught with challenges that must be addressed. The systemic delivery of miRNA mimics or antagomirs requires refined vehicles to ensure stability in circulation, prevent rapid renal clearance, and avoid potential immunogenicity [181]. Furthermore, off-target effects remain a significant concern, as a single miRNA can regulate hundreds of transcripts. A critical hurdle will be to achieve tissue- and cell-type-specific delivery to maximize on-target efficacy while mitigating unintended consequences in healthy tissues [178]. Ultimately, successful clinical applications will likely depend on the development of robust companion diagnostics to stratify patients based on tumor- or tissue-specific miR-483 expression levels and its associated network activity, indicating potential for a personalized medicine approach.

In summary, miR-483-based therapies remain preclinical. However, the mechanistic rationale and compelling preclinical data underscore its potential as an ‘ideal’ therapeutic target. Current research efforts are focused on bridging this translational gap, aiming to leverage miR-483 mimics to combat drug resistance and inhibitors to treat aggressive primary tumors.

## 5. Discussion

This review integrates research from 146 studies to explain how microRNA-483 exerts opposing functions across different diseases. By mapping its upstream regulators and downstream targets, we reveal that miR-483 acts as an oncogene in cancer by suppressing tumor suppressors, yet protects cardiac, pulmonary, and metabolic tissues from stress and fibrosis [177]. This paradox reflects miR-483’s control of core cellular pathways: TGF-β, MAPK, and apoptosis. Notably, TGF-β and MAPK pathways were specifically highlighted not only because they are frequently validated targets, but more importantly, because they represent the core, conserved signaling hubs modulated by miR-483 across diverse disease contexts, functioning as the key molecular switches through which miR-483 exerts its context-dependent activities. Their functional outputs depend on tissue-specific cofactors and target gene subsets [73,125]. For example, miR-483 targets the renin–angiotensin–aldosterone system exclusively in cardiovascular disease, defining its blood pressure regulatory role [138]. This context-dependent activity positions miR-483 as a molecular rheostat that adjusts universal signaling cascades to produce tissue-appropriate outcomes.

The functional plasticity of miR-483, oncogenic in tumors, protective elsewhere, likely arises from a complex interplay of transcriptional regulation and post-transcriptional interactions. At the transcriptional level, regulation is both *IGF2*-dependent and -independent. As an intronic miRNA within *IGF2*, basal miR-483 levels are co-transcribed with *IGF2* driven by growth factor signaling [34]. This “coupled” mode relies on the shared epigenetic status of the IGF2 locus, such as methylation at the DMR0 region recognized by KLF4, or repression by the ZBED6-GCTCGC motif interaction [34,50]. However, in pathological contexts such as tumorigenesis, an alternative, independent transcriptional program is activated via an independent promoter of miR-483 itself [64,122]. For instance, the β-catenin/USF1 complex directly binds the intronic promoter in response to metabolic stress [47]. In parallel, KLF9 acts on upstream regulatory elements to modulate miR-483 levels in response to proliferative cues [49]. Such independent regulation enables the rapid elevation of miR-483 to thresholds required for apoptosis suppression or metabolic adaptation, thereby functionally dissociating its activity from the canonical IGF2 growth axis.

Mechanistically, miR-483 suppresses gene expression through canonical recognition of complementary sequences within target 3′-UTRs [116]. This specificity is determined by the seed region (nucleotides 2–8 at the 5′ end), whose base-pairing with target mRNAs recruits the RNA-induced silencing complex (RISC) to mediate degradation or translational repression [182,183]. The short seed length creates many potential matches, but the targets aren’t random, evolutionarily conserved seed matches indicate functionally relevant regulation [184]. Moreover, this seed-matching paradigm is subject to multiple regulatory layers. RNA-binding proteins (RBPs) can modulate target accessibility by binding adjacent to seed-match sites, either exposing or occluding them to confer context-specific selectivity [185,186]. Furthermore, competitive endogenous RNAs, including lncRNAs and circRNAs, sequester miR-483 through decoy binding sites, effectively titrating its availability for target repression [46]. The tissue-specific abundance of these ceRNAs, for instance, lncRNA *NEAT1* is high in osteosarcoma but low in testes, creates different activity thresholds for miR-483 [187]. Together, these layers of transcriptional and post-transcriptional control create the context-specific “interactome” that ultimately governs whether miR-483 acts as an oncomiR or a tissue protector [188].

This context-dependence explains expression heterogeneity of miR-483. Several factors contribute to this functional plasticity. While technical variability between studies, such as different miRNA quantification and normalization methods, can play a role, the primary driver is the biological context itself. For example, the genetic status of TP53 fundamentally alters the downstream consequences of miR-483-mediated repression [119]. In p53-wildtype cells, miR-483-3p suppression of *MDM4* may enhance p53 activity and apoptosis, whereas in p53-mutant tumors, the same interaction becomes irrelevant, shifting the net effect of miR-483 toward oncogenicity via the repression of tumor suppressors like *PTEN* and *SMAD4* [119]. Similarly, the epigenetic status of the *IGF2* locus, particularly its imprinting, dictates the baseline expression of miR-483 and can fundamentally alter its functional impact, yet this is rarely addressed in functional studies [42].

Therefore, to truly understand the role of miR-483, a comprehensive, function-centric network model is not just helpful, but necessary. Such a framework allows us to place individual findings into a broader biological context and begin to unravel the logic behind its context-dependent activity. This network perspective addresses that miR-483 functions as a critical signal transducer that both responds to pathological stimuli and actively drives disease progression. Initial perturbations, such as hypoxia or inflammatory signaling, alter miR-483 expression, which then propagates these signals through extensive downstream target networks, ultimately establishing self-reinforcing pathological cascades. This bidirectional functionality transforms miR-483 from a mere biomarker into an active regulatory hub that amplifies disease phenotypes. Such mechanistic understanding has direct therapeutic implications: targeting miR-483 could disrupt these feedback loops at their nexus rather than addressing isolated downstream effects. This integrative framework thus shifts the research paradigm from correlative associations toward mechanistic interventions, offering more precise therapeutic opportunities.

Clinically, miR-483 shows promise as both a biomarker and therapeutic target. Circulating miR-483-5p is elevated in adrenocortical carcinoma, enabling diagnosis and recurrence monitoring [173]. Therapeutically, synthetic inhibitors could suppress miR-483 in cancers where they are overexpressed, while mimics could restore tumor-suppressive activity in drug-resistant lung cancer [189,190]. However, realizing this potential requires advances in tissue-specific delivery, such as antibody-conjugated nanoparticles or engineered exosomes.

Despite these advances, there remain some gaps that could be fulfilled in future research. For instance, utilizing single-cell RNA sequencing combined with spatial profiling can map miR-483 expression across tumor or fibrotic tissue heterogeneity, clarifying cell-type-specific roles [191]. Furthermore, employing CRISPR-Cas9 screening with a library targeting the 3′-UTRs of the predicted miR-483 targetome would systematically validate functional targets in different disease models and uncover context-dependent essential targets [185]. It is also crucial to investigate the upstream ‘code’ by characterizing the epigenetic landscape of the IGF2/miR-483 locus in various diseases to understand the signals that trigger IGF2-independent transcription [192]. Finally, developing safe, cell-type-specific delivery vehicles is essential for clinical translation [181]. Addressing these challenges will be essential for realizing the diagnostic and therapeutic promise of miR-483.

## 6. Conclusions

This review establishes miRNA-483 as a key regulator whose biological impact is dictated by its cellular context. By connecting its complex upstream regulators with its diverse downstream targets, we move beyond a fragmented, disease-by-disease perspective. Our central finding is that miR-483 can act as a potent cancer promoter in one context and a guardian of tissue stability in another. This dual role is explained by its ability to modulate a core set of universal pathways that are fine-tuned by tissue-specific interactions. This adaptability is rooted in a multi-layered regulatory network of transcriptional controls and post-transcriptional buffering. This system positions miR-483 as a highly sensitive rheostat, capable of translating diverse physiological and pathological signals into precise functional outcomes.

This integrated understanding underscores the clinical potential of miR-483. Its consistent dysregulation across numerous diseases establishes it as a robust biomarker for diagnosis and prognosis, while its central role in driving disease progression makes it a promising therapeutic target. The primary challenge for the field is therefore to translate this mechanistic knowledge into clinical applications. This will require the development of strategies to selectively modulate miR-483 activity in a tissue- and disease-specific manner. Successfully harnessing the context-dependent nature of this powerful miRNA is key to unlocking a new generation of diagnostic tools and therapeutic interventions.

## Figures and Tables

**Table 1:** Upstream Regulator-miR-483 Interaction Summary Table. The table summarizes factors demonstrated to modulate miR-483 expression or availability. Regulation Mode specifies the mechanism: transcriptional activation/repression by TFs, or physical Sequestration by lncRNAs/circRNAs. Representative downstream targets and associated disease phenotypes resulting from these upstream interactions are included. Target states are noted as (repressed) for direct inhibition via post-transcriptional repression, or (derepressed) for released expression.

Upstream Regulator	Regulation Mode	miRNA Isoform	Downstream Target/Pathway	Functional Effect	Related Disease/State	Source
**Transcription Factor Regulation**						
CTNNB1/USF1	Activation	miR-483	*BBC3*/*PUMA* (repressed)	Enhances tumor progression and chemoresistance	Cancers	[42,44,47]
EGR1	Activation	miR-483	Not Specified	Associated with tumor progression, poor prognosis	Human carcinomas	[48]
KLF4	Activation	miR-483	*CTGF* (derepressed)	Suppress EMT	Kawasaki disease	[34]
KLF9	Activation	miR-483-3p	*MMP9* (repressed)	Inhibits cancer cell proliferation and invasion	Testicular seminoma	[49]
ZBED6	Repression	miR-483	PI3K-Akt signaling	Represses IGF2 and miR-483 expression	Muscle hypertrophy, Cancers	[50]
WT1	Repression	miR-483	ERK signaling	Induce mesenchyme differentiation	Cancers	[42]
**Circular RNA Regulation**						
circ_0000006	Sequestration	miR-483-5p	*KDM2B* (derepressed)	Promotes VSMC proliferation & phenotypic switching	Aortic dissection	[51]
circ_0122153	Sequestration	miR-483-3p	RAAS signaling	Elevated blood pressure	Essential hypertension	[52]
circ_0123190	Sequestration	miR-483-3p	*APLNR* (repressed)	Exacerbates renal inflammation and fibrosis	Lupus nephritis	[46]
circ-ASAP1	Sequestration	miR-483-3p	mTOR/MAPK signaling	Contributes to cardiac dysfunction	Cirrhotic cardiomyopathy	[53]
circ_0006859	Sequestration	miR-483-3p	*EFNA2*, *DOCK3* (derepressed)	Inhibits osteogenic differentiation of BMSCs, promotes bone loss	Osteoporosis (post-menopausal)	[54]
circUTRN24	Sequestration	miR-483-3p	*IGF1* (derepressed)	Influences fibrosis progression, HSC autophagy	Biliary atresia (liver fibrosis)	[55]
circZNF609	Sequestration	miR-483-3p	*CDK6* (derepressed)	Promotes the proliferation and migration of gastric cancer	Gastric cancer	[56]
circRNA_02767	Sequestration	miR-483-3p	*GFAP* (derepressed)	Alleviates central sensitization, reduces neuropathic pain	Chronic inflammatory visceral pain/Neuropathic pain	[57]
**Long Non-coding RNA Regulation**						
*NEAT1*	Sequestration	miR-483-3p	*STAT3* (derepressed)	Promotes EMT, metastasis	Osteosarcoma	[58]
*NEAT1*	Sequestration	miR-483-3p	*UBE2C* (derepressed)	Associated with progression, biomarker potential	Prostate cancer	[59]
*NR2F1-AS1*	Sequestration	miR-483-3p	*IGF1* (derepressed)	Fosters azacitidine resistance	Acute myeloid leukemia	[60]
*NR2F1-AS1*	Sequestration	miR-483-3p	*FOXA1* (derepressed)	Drives malignant progression	Osteosarcoma	[61]
*LINC00662*	Sequestration	miR-483-3p	*SOX3* (derepressed)	Promoted tumor proliferation and invasiveness	Glioma	[62]
*MIR4500HG003*	Sequestration	miR-483-3p	*MMP9* (derepressed)	Enhances metastasis	Triple-negative breast cancer	[63]
*SNHG11*	Sequestration	miR-483-3p	*CTNNB1*, *ATG12* (derepressed)	Facilitates oncogenic autophagy	Gastric cancer	[64]
*BCAR4*	Sequestration	miR-483-3p	*RAB5C* (derepressed)	Enhances chemotherapy resistance (Oxaliplatin)	Colorectal cancer	[65]
*TTC39A-AS1*	Sequestration	miR-483-3p	*MTA2* (derepressed)	Promoted proliferation and metastasis	Breast cancer	[45]
*LINC00908*	Sequestration	miR-483-5p	*TSPYL5* (derepressed)	Inhibits proliferation and metastasis	Prostate Cancer	[66]
*MEG3*	Sequestration	miR-483-3p	*ERp29* (derepressed)	Promotes proliferation and migration	Hepatocellular carcinoma	[67]
*H19*	Sequestration	miR-483-5p	*DUSP5* (derepressed)	Mitigates mechanical stress-induced cartilage degradation	Developmental dysplasia of the hip/Osteoarthritis	[68]
*H19*	Sequestration	miR-483-3p	Wnt/β-catenin signaling	Promotes osteogenic differentiation	Osteogenic differentiation	[69]
*SNHG14*	Sequestration	miR-483-5p	*HDAC4* (derepressed)	Exacerbates renal tubular damage, inflammation, fibrosis	Diabetic kidney disease	[70]
*MALAT1*	Sequestration	miR-483-3p	hs-CRP (derepressed)	Enhances inflammation	Acute cerebral infarction	[71]
*DBH-AS1*	Sequestration	miR-483-5p	HCAEC function (proliferation, apoptosis, inflammation)	Influences endothelial dysfunction, predicts CV events	Type 2 diabetes mellitus/Coronary heart disease	[72]
*SNHG29*	Sequestration	miR-483-3p	*CBL* (derepressed)	Drives progression	Chronic myeloid leukemia	[73]
*MPRL*	Sequestration	miR-483-5p	*FIS1* (derepressed)	Enhances cisplatin sensitivity	Tongue squamous cell carcinoma	[74]
miR-483-5p	Activation	miR-483-5p	Binding *IGF2*/*H19* enhancer	Promote HCC malignant progression	Hepatocellular carcinoma	[75]

**Table 2:** Key validated miR-483 targets across six disease categories.

**Disease Category**	**miR-483 Isoform**	**Validated Targets**
Neoplasm	miR-483-3p	*BBC3*, *PUMA*, *MMP9*, *CCNE1*, *PTEN*, *CDK4*, *CDK6*, *CDC25A*, *RB1*, *MDM4*, *DKK3*, *SMAD4*, *SMAD2*, *ITGB3*, *IGF1*, *BRCA1*
	miR-483-5p	*MAPK1*, *ERK2*
Cardiopulmonary	miR-483-3p	*AGT*, *ACE*, *ACE2*, *AGTR2*, *VEZF1*
	miR-483-5p	*MAPK1*, *MAPK3*, *ERK2*, *ERK1*, *ET-1*, *TGFB1*, *TGFBR2*, *SMAD2*, *ROCK1*, *TIMP2*, *PDGFB*, *TNFSF8*, *CTGF*, *PCSK9*
Metabolic	miR-483-3p	*IGF1*, *IGF1R*
	miR-483-5p	*PDX1*, *MAFA*, *MAPK1*, *ERK2*, *TIMP2*, *HDAC4*, *ALDH1A3*, *PCSK9*
Immune-mediated	miR-483-3p	*CTGF*, *HIPK2*, *APLNR*, *CD81*, *RNF5*, *IGF1*
	miR-483-5p	*SRSF4*, *HDAC2*, *COL1A1*, *FLI1*
Nervous System	miR-483-3p	*XPO1*
	miR-483-5p	*ERK1*, *ERK2*, *GPX3*, *PGAP2*
Musculoskeletal	miR-483-3p	*DKK2*, *SMAD4*, *IGF1*, *IGF2*, *SRF*
	miR-483-5p	*SATB2*, *RPL31*, *MATN3*, *DUSP5*, *IGF2*, *MAPK1*, *MAPK3*, *ERK2*, *ERK1*, *NOTCH3*, *SRF*

## Supplementary Materials

The following supporting information can be downloaded at: https://www.mdpi.com/article/10.3390/biom15121707/s1, Supplemental method: Bioinformatic analysis of miR-483 regulatory networks in cancer; Figure S1: Expression landscape of miR-483-3p and miR-483-5p across multiple cancer types in TCGA; Figure S2: Cancer-specific regulatory sub-networks of miR-483 in TCGA cohorts; Figure S3: Pan-cancer survival analysis of miR-483 expression; Table S1: Role of miR-483 in various diseases and associated target genes.
