# Supplementary material for: Integrative Regulatory Networks of MicroRNA-483: Unveiling Its Systematic Role in Human Diseases and Clinical Implications
Source: Biomolecules. 2025 Dec 7;15(12):1707. doi: 10.3390/biom15121707 (PMC12730746; doi:10.3390/biom15121707)
Supplement: Supplementary file 1 [file biomolecules-15-01707-s001.zip › 2_miR-483_review_Supplementary_proofreaded.pdf]

SUPPLEMENTARY DATA

Integrative Regulatory Networks of microRNA-483: Unveiling Its Systematic Role in Human Diseases and Clinical Implications

SUPPLEMENTAL METHOD

S1. Bioinformatic Analysis of miR-483 Regulatory Networks in Cancer

S1.1. TCGA Data Acquisition and Processing

To conduct a pan-cancer analysis of miR-483's role, we obtained Level 3 miRNA mature strand expression data (RNA-Seq) and gene expression data (RNA-Seq) from The Cancer Genome Atlas (TCGA) database via the USCS Xena browser (https://xenabrowser.net). We included cancer types in our analysis only if they showed statistically significant differential expression of hsa-miR-483-3p or hsa-miR-483-5p between tumor and normal tissues (Wilcoxon rank sum test, $p < 0.05$). This selection process resulted in 15 TCGA cancer cohorts for sub-network construction.

S1.2. Identification of Cancer-Specific Differentially Expressed Genes (DEGs)

For each of the 15 selected cancer types, we performed differential expression analysis on the gene expression data to identify DEGs between tumor and adjacent normal tissues. To ensure robustness, we employed three widely used R packages: DESeq2 [1], edgeR [2], and limma-voom [3]. A gene was considered a DEG if it met the criteria of a p-value < 0.05 and a |Log2 Fold Change| > cutoff. The Log2FC cutoff was dynamically determined for each dataset to capture the most significant changes, calculated as: mean (|Log2FC of all genes|) + 2 * standard deviation (|Log2FC of all genes|). The final list of DEGs for each cancer type was generated by taking the union of the results from the three R packages.

S1.3. Construction of Cancer-Specific TF-miR-483-mRNA Sub-Networks

The cancer-specific regulatory sub-networks were constructed by identifying the intersection between two sets of genes: The list of cancer-specific DEGs identified in the previous step; the global, experimentally validated miR-483 regulatory network described in the main manuscript (Figure 2), here we focused on upstream transcription factors and downstream mRNA targets. An additional set of TargetScan [4] predicted targets was compiled to provide a comprehensive downstream regulatory landscape. Cumulative weighted context++ scores of less than -0.4 were used as the threshold for predicted target selection regarding miR-483-3p and miR-483-5p. Upstream TFs for hsa-miR-483 were identified from two sources: experimentally validated interactions reported in literature and TF-miRNA regulatory pairs archived in TransmiR v2.0, a database compiling ChIP-seq evidence [5]. Putative TFBS were identified using the MATCH tool in TRANSFAC [6]; predicted TF families were then deconvoluted into individual TFs using Ensembl [7]. This intersection ensures that each sub-network only contains regulators and targets of miR-483 that are also significantly dysregulated in that specific cancer type. The resulting sub-networks were visualized using Cytoscape (version 3.9.1) [8].

S1.4. Pan-Cancer Survival Analysis

To assess the prognostic value of miR-483, we utilized the Kaplan-Meier plotter (KM plotter) database (http://kmplot.com), a meta-analysis tool for biomarker validation. We analysed the correlation between miR-483 expression and Overall Survival (OS) across various TCGA cancer cohorts. Patients were stratified into high- and low-expression groups based on the median expression level of miR-483. The statistical significance of the difference in survival between the two groups was calculated using the log-rank test. A p-value < 0.05 was considered statistically significant [9].

SUPPLEMENTAL FIGURE

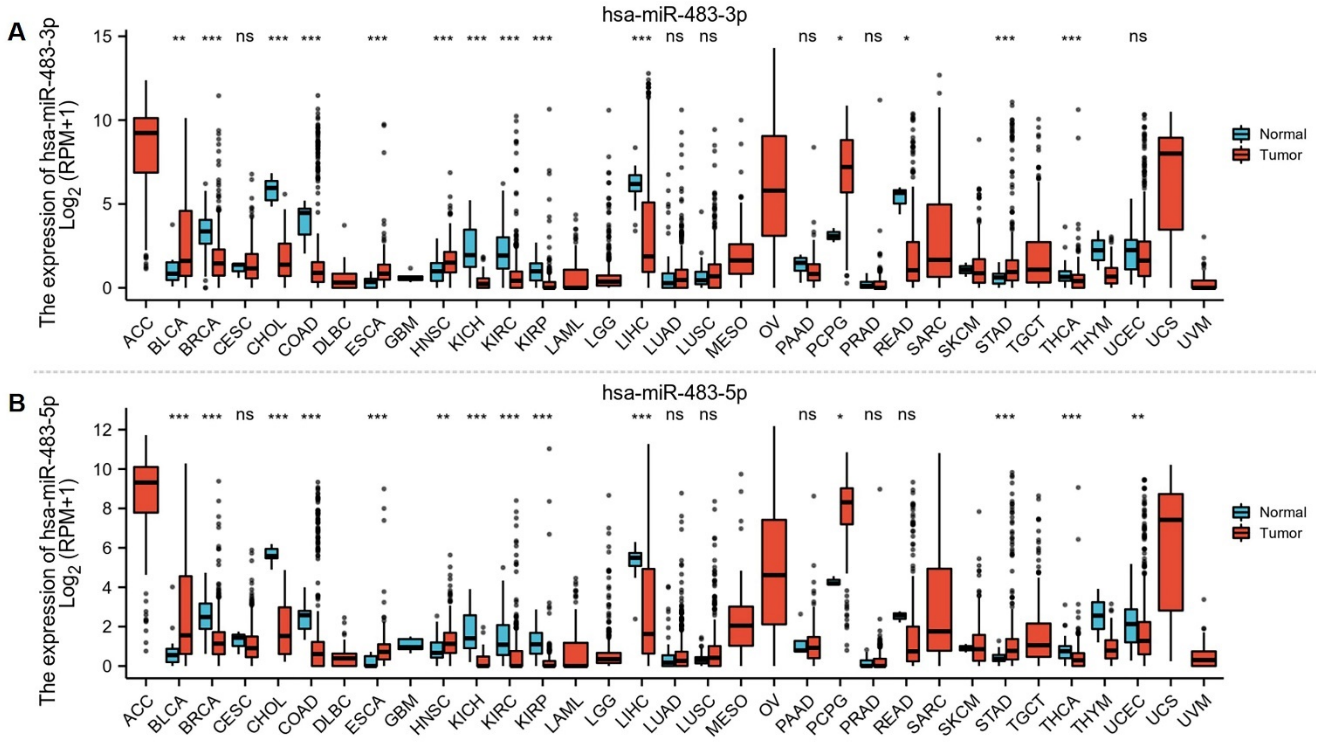

Supplementary Figure S1. Expression landscape of miR-483-3p and miR-483-5p across multiple cancer types in TCGA. Boxplots comparing the expression levels of (Top) hsa-miR-483-3p and (Bottom) hsa-miR-483-5p in tumor tissues (red) versus adjacent normal tissues (blue). Expression data are presented as Log2-transformed Reads Per Million (RPM+1). Statistical significance of the difference between tumor and normal samples was determined using the Wilcoxon rank-sum test. Asterisks denote the level of significance ($p < 0.05$, *$p < 0.01$, **$p < 0.001$); 'ns' indicates no significant difference. This pan-cancer analysis highlights the profound expression heterogeneity of miR-483. Notably, there is significant intra-tumor variability, with a wide distribution of expression levels among patients within the same cancer type. Even in cancers with a statistically significant trend (e.g., upregulation in BRCA), the expression ranges between tumor and normal tissues often overlap. This high degree of patient-to-patient heterogeneity underscores the limitations of using expression levels alone to infer function and validates our review's function-centric approach to understanding the core biological roles of miR-483.

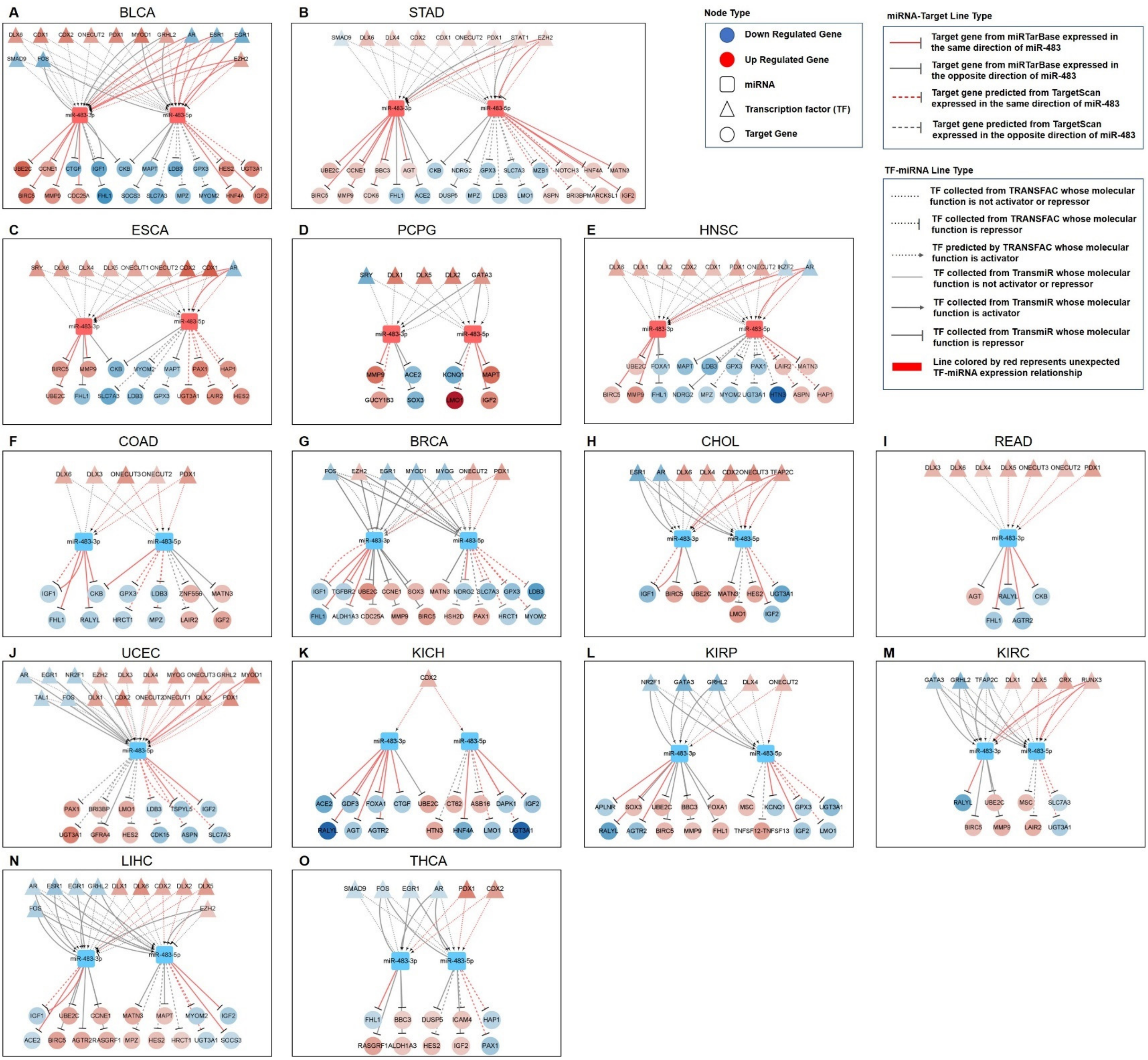

Supplementary Figure S2. Cancer-specific regulatory sub-networks of miR-483 in TCGA Cohorts. This figure illustrates the heterogeneity of the miR-483 regulatory network across 15 different cancer types from The Cancer Genome Atlas (TCGA). Sub-networks were constructed by intersecting cancer-specific differentially expressed genes with the global miR-483 interactome. (A-E) show networks for cancers where miR-483 is generally upregulated. (F-O) show networks for cancers where miR-483 is generally downregulated. For each cancer-specific sub-network, red and blue nodes denote upregulated and downregulated genes, with colour intensity reflecting expression magnitude. Edge styles distinguish data sources: for TFs, dotted lines for TRANSFAC-predicted TFs, solid lines for experimentally derived TFs (TransmiR); for targets, dashed lines for TargetScan predicted targets, and solid lines denote literature-validated targets from miRTarBase. TF symbols follow UniProt functional annotations: arrows for activators, suppression bars for repressors, and neutral symbols for unclassified factors. All downstream genes carry suppression bars, consistent with canonical miRNA-mediated repression. (A) BLCA: Bladder Cancer; (B) STAD: Stomach Cancer; (C) ESCA: Esophageal Cancer; (D) PCPG: Pheochromocytoma and Paraganglioma; (E) HNSC: Head and Neck Cancer; (F) COAD:

Colon Cancer; (G) BRCA: Breast Cancer; (H) CHOL: Bile Duct Cancer; (I) READ: Rectal Cancer; (J) UCEC: Endometrioid Cancer; (K) KICH: Kidney Chromophobe; (L) KIRP: Kidney Papillary Cell Carcinoma; (M) KIRC: Kidney Clear Cell Carcinoma; (N) LIHC: Liver Cancer; (O) THCA: Thyroid Cancer.

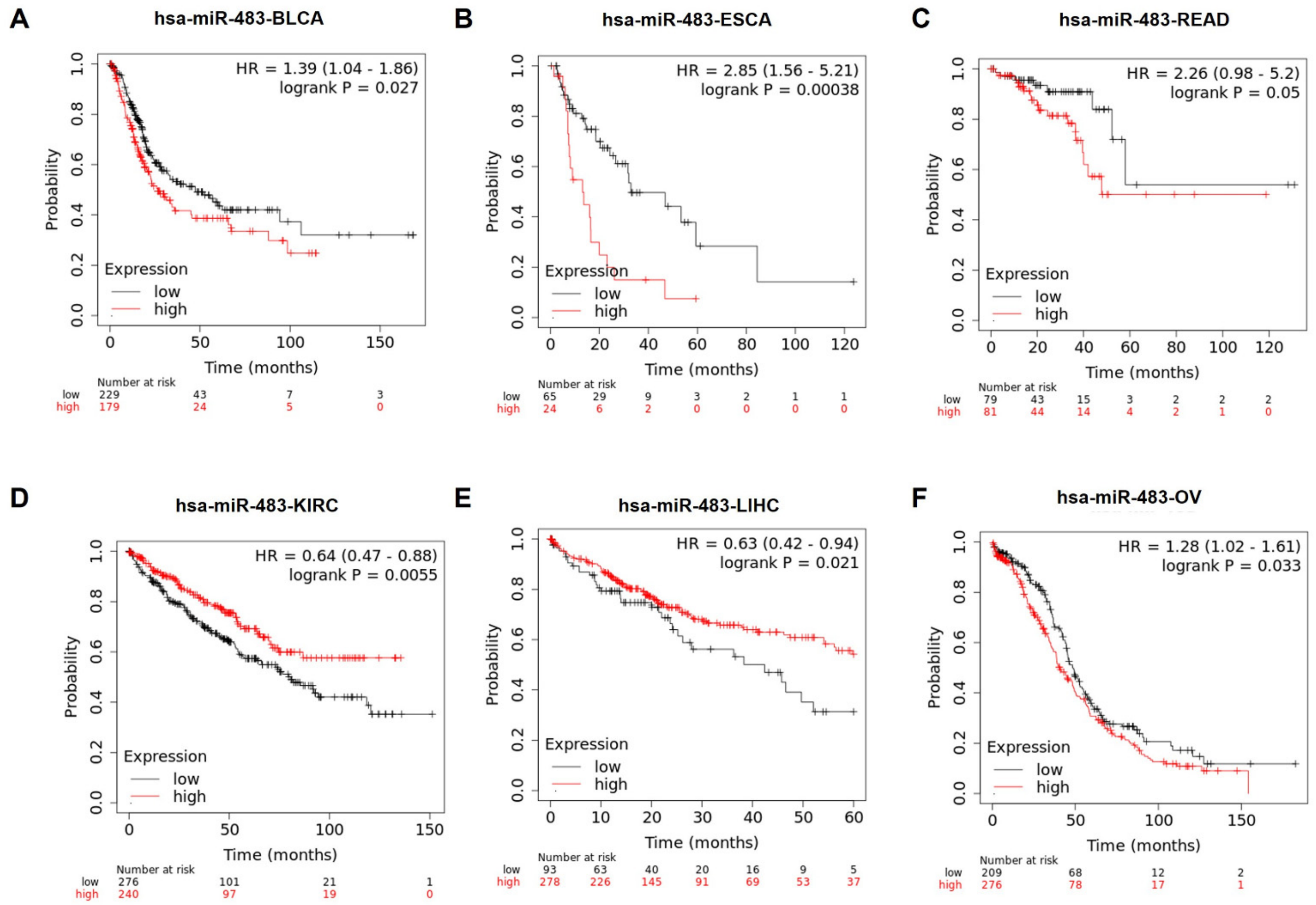

Supplementary Figure S3. Pan-cancer survival analysis of miR-483 expression. Kaplan-Meier plots illustrating the prognostic significance of miR-483 expression on Overall Survival (OS) in 6 different cancer types from the TCGA database. For each cancer, patients were stratified into high-expression (red line) and low-expression (black line) groups based on the median expression level. Hazard Ratios (HR) with 95% confidence intervals and log-rank p-values are displayed on each plot. A p-value < 0.05 was considered statistically significant, indicating a significant difference in survival between the two groups. This analysis highlights the context-dependent prognostic role of miR-483, where high expression is associated with poor prognosis in some cancers but better prognosis in others.

SUPPLEMENTAL TABLE

Supplementary Table S1. Role of miR-483 in various diseases and associated target genes

Diseases	Family members	Expression	Target genes	Tissues/Cell lines	Refs	Function
**Cancers**						
Colon cancer	miR-483-5p	down	*TRAF1*	SW480, HCT8 cells	[10]	inhibit proliferation
	miR-483-3p	up	*ANAPC11, MDM4, UBE2C, EI24*	CaCo-2, HCT116 cells	[11], [12]	tumor promoter
Colorectal cancer	miR-483-5p, miR-483-3p	down	*CKB, DKK3, FAM171B, RAB5C*	LS174T, SW620, WiDR, LvM3a, LvM3b, HCT116/L cells	[13], [14], [15], [16]	tumor suppressor, oxaliplatin resistance
	miR-483-5p, miR-483-3p	up	*IGF2, DLC1, ATP5G1, CYC1, BBC3, PUMA, NDRG1*	serum, HCT116 cells	[17], [18], [19], [20]	tumor promoter
Non-small cell lung cancer	miR-483-5p	up	*RHOGDI1, ALCAM, RBM5*	A549, PC9 cells	[21], [22], [23]	promotes EMT, antiapoptosis
	miR-483-3p	down	*INTEGRIN B3*	HCC827, H1975, A549, H292, H1299 cells	[24]	inhibit proliferation
Osteosarcoma	miR-483-3p	down	*FOXA1, STAT3*	U2OS, MG-63, Saos-2, HOS, osteosarcoma tissue	[25] , [26]	tumor suppressor
Hepatocellular carcinoma	miR-483-5p	down	*RAI16, PPARα, TIMP2*	HepG2, Huh7 cells	[27], [28]	tumor suppressor
	miR-483-5p, miR-483-3p	up	*SOCS3, ALCAM, IGF2, BBC3, PUMA, ERP29*	SK-Hep1, SMMC-7721, Huh7, Hepa1-6, Hep3B, HepG2, SNU-449 cells, serum	[29], [30], [31], [32], [33], [34]	tumor promoter

Breast cancer	miR-483-3p	down	*SOX3, METTL3, HDAC8, CCNE1, MMP9*	MCF-10A, HBL-100, MCF-7, T47D, MDA-MB-231 cells	[35], [36], [37], [38], [39]	tumor suppressor
Adrenocortical carcinoma	miR-483-5p, miR-483-3p	up	*NDRG2, PUMA*	NCI-H295R, SW13 cells	[40], [41]	tumor promoter
Anaplastic thyroid cancer	miR-483-3p	up	*PARD3*	FRO, 8505C, HTH7 cells	[42]	tumor promoter
Neuroblastoma	miR-483-3p	up	*PUMA*	SH-SY5Y, SK-N-BE, IMR-32 cells	[43]	tumor promoter
Glioma	miR-483-5p, miR-483-3p	down	*ERK1, SOX3*	LN18, LN229, U87, U251, SHG44 cells	[44], [45,46]	tumor suppressor
Meningioma	miR-483-5p	up	*IGF-2*	Meningioma tumor samples	[47]	tumor promoter
Nasopharyngeal carcinoma	miR-483-5p	up	*DAPK1, EGR3*	CNE-1, 5-8F cells	[48], [49]	tumor promoter
Esophageal cancer	miR-483-5p	up	*KCNQ1*	EC cells	[50]	tumor promoter
Esophageal squamous cell carcinomas	miR-483-5p	up	*HNF4A*	SHEEC, SHEE cells	[51]	tumor promoter
	miR-483-3p	up	*EI24*	EC109, EC9706, TE-1, ESCC tissue	[52], [53]	tumor promoter
Prostate cancer	miR-483-5p	up	*RBM5, TSPYL5*	DU-145, PC-3 cells	[54], [55]	tumor promoter
	miR-483-3p	up	*PUMA, BAK1*	PC-3 cells	[56]	
Ovarian carcinoma	miR-483-3p	up	*PRKCA*	IGROV-1 cells	[57]	induce drug resistance

Epithelial ovarian cancer	miR-483-5p	up	*TAOK1*	EOC tissue	[58]	Predicts Chemotherapy Resistance
Pancreatic cancer	miR-483-3p	up	*DPC4, SMAD4*	pancreatic cancer tissue	[59]	tumor promoter
Pancreatic ductal adenocarcinoma	miR-483-3p	up	*SMAD4*	blood, PDAC tissue	[60]	diagnostic biomarker
Seminoma	miR-483-3p	down	*MMP9*	SEM tissue, Tcam-2	[61]	tumor suppressor
Paragangliomas	miR-483-5p	up	*ALCAM*	PPGL tissue, serum	[62]	metastases
Gastric cancer	miR-483-3p	down	*CDK6, OGT, CTNNB1*	GES-1, SGC-7901, HGC-27, AGS, MKN-45, BGC-823, MKN28, SNU16, GC tissue	[63], [64], [65]	tumor suppressor
Wilms' tumor	miR-483-5p	down	*MKNK1*	GHINK-1 cells	[66]	inhibit proliferation
	miR-483-3p, miR-483-5p	up	*PTEN, IGF2*	Wit49 cells, frozen Wilms’ tumor	[67], [68]	tumor promoter
Malignant mesothelioma	miR-483-3p	up	*RB1*	Malignant Mesothelioma tissue	[69]	tumor promotor
Chronic Myeloid Leukemia	miR-483-3p	down	*CBL*	CML patient blood samples	[70]	tumor promotor
Acute Monocytic Leukemia	miR-483-3p	down	*IGF1*	THP-1 cells	[70]	Sensitizes to azacitidine
Squamous cell carcinomas	miR-483-3p	down	*API5, RAN, BIRC5, CDC25A*	CAL27, CAL33, CAL60 cells	[71]	tumor suppressor

Tongue squamous cell carcinoma	miR-483-5p	up	*FIS1*	HSC-3 cells	[72]	inhibit mitochondrial fission and cisplatin sensitivity
Multiple myeloma	miR-483-5p	up	*TIMP2*	Bone marrow mononuclear cells	[73]	promote malignant progression
**Infectious Diseases**						
Influenza	miR-483-3p	up	*RNF5, CD81*	bronchoalveolar lavage fluid exosomes, MLE-12 cells	[74]	Regulates the Innate Immune Response
Sepsis-Induced Intestinal Injury	miR-483-3p	up	*HIPK2*	Human intestinal epithelial NCM460 cells	[75]	Promotes apoptosis/cytotoxicity
Severe pneumonia	miR-483-3p	up	*IGF-1*	MRC-5 cells	[76]	promoted the development of severe pneumonia
**Inflammatory & Autoimmune Diseases**						
Systemic sclerosis	miR-483-5p	up	*COL4A1, COL4A2, FLI1*	serum	[77]	potential driver of fibrosis
Lupus nephritis	miR-483-3p	up	*APLNR*	renal tissue	[78]	Regulate Renal fibrosis
Henoch-Schonlein purpura	miR-483-5p	down	*IL6*	PBMC cells	[79]	correlated with interleukin-6
Rheumatoid Arthritis	miR-483-5p	up	*SRSF4*	Peripheral blood exosomes	[80]	Potential exosomal biomarker
Periodontitis	miR-483-5p	up	*COL1A1*	Macrophage-derived ABs	[81]	inhibits osteoblast differentiation

Severe Acute Pancreatitis	miR-483-5p	down	*HDAC2*	BEAS-2B cells	[82]	Regulate disease progression
Kawasaki disease	miR-483-3p	down	*CTGF*	Endothelial cells	[83]	associated with endothelial-mesenchymal transition
**Metabolic & Endocrine Diseases**						
Diabetes	miR-483-3p, miR-483-5p	up	*GDF3, ALDH1A3, PDX1, MAFA*	adipose tissue, pancreatic beta cells	[84], [85], [86]	increasing susceptibility to metabolic disease
Diabetic nephropathy	miR-483-5p, miR-483-3p	up	*HDCA4, MAPK1, TIMP2, IGF-1*	serum, urine, HK-2 cells	[87], [88], [89]	promotes the progression of diabetic nephropathy
Hypercholesterolemia	miR-483-5p	up	*PCSK9*	HepG2 cells	[90]	ameliorate hypercholesterolemia
Alcoholic hepatitis	miR-483-3p	down	*BRCA1*	liver biopsies	[91]	regulate liver mallory-denk bodies formation
Diabetic Retinopathy	miR-483-5p	down	*IGF-1R*	ARPE-19, BMSC cells	[92]	Protects from HG-induced apoptosis
**Cardiovascular & Related Diseases**						
Hypertension	miR-483-3p	down	*AGT, ACE1, ACE2, AGTR2*	HEK-293, HEK-AT1R, HEK-AT2R, RASMC, RASMC-AT1R, RASMC-AT2R, HASMC cells	[93]	negative regulator of steady-state levels of RAS components

Pulmonary arterial hypertension	miR-483-3p, miR-483-5p	down	*TGF-b, TGFBR2, SMAD2, ROCK1, ET-1, IL1B*	serum, pulmonary endothelial cells	[94]	amelioration of experimental pulmonary hypertension
Atherosclerosis	miR-483-5p	up	*TIMP2*	HUVEC cells	[95]	Promotes injury
Calcific aortic valve disease	miR-483-3p	down	*UBE2C*	HAVEC cells	[96]	potential therapeutics of calcific AV disease
Deep vein thrombosis	miR-483-3p, miR-483-5p	up	*SRF, MAPK1*	Endothelial progenic cells, HUVEC cells	[97], [98]	endothelial progenitor cells dysfunction, anti-inflammatory
Coronary plaque rupture	miR-483-3p	up	*IGF-1*	H9C2 cells	[99]	potential biomarkers for heart complications
Ischemic heart disease	miR-483-5p	down	*SRF*	HUVEC cells	[100]	control angiogenesis
	miR-483-3p	up	*OGT, SLC1A3, SATB1*	postischemic LV samples	[101]	inducing cell death
Acute myocardial infarction	miR-483-5p, miR-483-3p	up	*IGF-1, MAPK3*	H9C2, AC16, plasma	[102]	potential therapeutic target for AMI
Aggravated endothelial injury	miR-483-3p	up	*VEZF1*	M2MFs, aortic wall, HAEC cells	[103]	regulate endothelial integrity
Postcardiac Arrest Brain Injury	miR-483-5p	down	*TNFSF8*	PC12 cells, Hippocampal samples	[104]	neuroprotective

Myocardial ischemia reperfusion	miR-483-3p	up	*MDM4*	H9C2 cells	[105]	inhibition ameliorates myocardial ischemia
Chronic obstructive pulmonary disease	miR-483-5p	down	*TGFB1*	COPD samples	[106]	promote proliferation
Sepsis-induced acute lung injury	miR-483-5p	up	*PIAS1*	pulmonary microvascular endothelial cells	[107]	aggravate inflammation and apoptosis
**Bone Related Diseases**						
Osteoarthritis	miR-483-5p	up	*TIMP2, MATN3*	osteoarthritis cartilage, articular cartilage, chondrocytes	[108]	pathogenesis of OA
Osteoporosis	miR-483-5p	up	*MAPK1, SMAD5*	fresh femoral neck trabecular bone-fractured bones, serum, osteoporotic tissue, BMSCs	[109], [110]	pathogenesis of osteoporosis, inhibits osteogenesis
Postmenopausal osteoporosis	miR-483-5p	up	*SATB2*	MC3T3-E1 cells	[111]	pathogenesis of osteoporosis
Intervertebral disk degeneration	miR-483-3p	up	*CTNNB1*	human nucleus pulposus cells	[112]	regulation of Wnt pathway
Developmental dysplasia of the hip	miR-483-5p	up	*DUSP5*	intermittent cyclic mechanical stress cells	[113]	regulate cartilage degradation
Rheumatoid arthritis	miR-483-3p	up	*IGF-1*	synovial tissue samples, fibroblast-like synoviocytes	[114]	promotes proliferation

Hypertrophic chondrocyte	miR-483-3p, miR-483-5p	up	*SMAD4*	human bone marrow-derived mesenchymal stem cells	[115]	suppress chondrogenic differentiation
**Regenerate System**						
Preeclampsia	miR-483, miR-483-5p	down	*IGF-1, STC2*	venous blood, umbilical cord blood, placental tissue	[116], [117]	regulate placental cellular activities during normal development and placental complications
Polycystic ovary syndrome	miR-483-3p, miR-483-5p	down	*IGF-1, SOCS3, SRF, NOTCH3, MAPK3*	cumulus cells of metaphase II oocytes, KGN	[118], [119], [120]	reducing insulin resistance, inhibit proliferation
**Neuron Related Diseases**						
Alzheimer's Disease	miR-483-5p	up	*ERK1, ERK2*	HDFN, EMEM	[121]	Lowers TAU Phosphorylation
	miR-483-3p	down	*XPO1*	PC12 cells	[122]	Improves learning and memory abilities
Anxiety	miR-483-5p	up	*PGAP2, GPX3, MACF1*	Amygdala	[123]	Reducing the stress
**Genetic Diseases**						
Duchenne muscular dystrophy	miR-483-5p	up	*IGF2*	serum	[124]	novel candidate biomarker
Multiple symmetric lipomatosis	miR-483-5p	up	*ERK1, ERK2*	subcutaneous adipose tissue	[125]	promote adipogenesis

Hirschsprung's Disease	miR-483-3p	down	*FHL1*	HSCR aganglionic colon tissues	[126]	suppressed cell migration and proliferation
	miR-483-5p	up	*GFRA4*	HSCR colonic tissues	[127]	promotes cell proliferation and invasion

References

1. Love, M.I.; Huber, W.; Anders, S. Moderated estimation of fold change and dispersion for RNA-seq data with DESeq2. *Genome Biol* **2014**, *15*, 550, doi:10.1186/s13059-014-0550-8.
2. Robinson, M.D.; McCarthy, D.J.; Smyth, G.K. edgeR: a Bioconductor package for differential expression analysis of digital gene expression data. *Bioinformatics* **2010**, *26*, 139-140, doi:10.1093/bioinformatics/btp616.
3. Law, C.W.; Chen, Y.; Shi, W.; Smyth, G.K. voom: Precision weights unlock linear model analysis tools for RNA-seq read counts. *Genome Biol* **2014**, *15*, R29, doi:10.1186/gb-2014-15-2-r29.
4. Lewis, B.P.; Burge, C.B.; Bartel, D.P. Conserved seed pairing, often flanked by adenosines, indicates that thousands of human genes are microRNA targets. *Cell* **2005**, *120*, 15-20, doi:10.1016/j.cell.2004.12.035.
5. Tong, Z.; Cui, Q.; Wang, J.; Zhou, Y. TransmiR v2.0: an updated transcription factor-microRNA regulation database. *Nucleic Acids Res* **2019**, *47*, D253-D258, doi:10.1093/nar/gky1023.
6. Wingender, E.; Chen, X.; Hehl, R.; Karas, H.; Liebich, I.; Matys, V.; Meinhardt, T.; Pruss, M.; Reuter, I.; Schacherer, F. TRANSFAC: an integrated system for gene expression regulation. *Nucleic Acids Res* **2000**, *28*, 316-319, doi:10.1093/nar/28.1.316.
7. Howe, K.L.; Achuthan, P.; Allen, J.; Allen, J.; Alvarez-Jarreta, J.; Amode, M.R.; Armean, I.M.; Azov, A.G.; Bennett, R.; Bhai, J.; et al. Ensembl 2021. *Nucleic Acids Res* **2021**, *49*, D884-D891, doi:10.1093/nar/gkaa942.
8. Tang, Y.; Li, M.; Wang, J.; Pan, Y.; Wu, F.X. CytoNCA: a cytoscape plugin for centrality analysis and evaluation of protein interaction networks. *Biosystems* **2015**, *127*, 67-72, doi:10.1016/j.biosystems.2014.11.005.
9. Gyorffy, B. Discovery and ranking of the most robust prognostic biomarkers in serous ovarian cancer. *Geroscience* **2023**, *45*, 1889-1898, doi:10.1007/s11357-023-00742-4.
10. Niu, Z.Y.; Li, W.L.; Jiang, D.L.; Li, Y.S.; Xie, X.J. Mir-483 inhibits colon cancer cell proliferation and migration by targeting TRAF1. *Kaohsiung J Med Sci* **2018**, *34*, 479-486, doi:10.1016/j.kjms.2018.04.005.
11. McCann, M.J.; Rotjanapun, K.; Hesketh, J.E.; Roy, N.C. Expression profiling indicating low selenium-sensitive microRNA levels linked to cell cycle and cell stress response pathways in the CaCo-2 cell line. *Br J Nutr* **2017**, *117*, 1212-1221, doi:10.1017/S0007114517001143.

12. Zhou, W.; Yang, W.; Yang, J.; Zhu, H.; Duan, L.; Wang, X.; Li, Y.; Niu, L.; Xiao, S.; Zhang, R.; et al. miR-483 promotes the development of colorectal cancer by inhibiting the expression level of EI24. *Mol Med Rep* **2021**, *24*, doi:10.3892/mmr.2021.12206.
13. Guo, J.; Yang, Z.; Zhou, H.; Yue, J.; Mu, T.; Zhang, Q.; Bi, X. Upregulation of DKK3 by miR-483-3p plays an important role in the chemoprevention of colorectal cancer mediated by black raspberry anthocyanins. *Mol Carcinog* **2020**, *59*, 168-178, doi:10.1002/mc.23138.
14. Liang, H.; Xu, Y.; Zhang, Q.; Yang, Y.; Mou, Y.; Gao, Y.; Chen, R.; Chen, C.; Dai, P. MiR-483-3p regulates oxaliplatin resistance by targeting FAM171B in human colorectal cancer cells. *Artif Cells Nanomed Biotechnol* **2019**, *47*, 725-736, doi:10.1080/21691401.2019.1569530.
15. Loo, J.M.; Scherl, A.; Nguyen, A.; Man, F.Y.; Weinberg, E.; Zeng, Z.; Saltz, L.; Paty, P.B.; Tavazoie, S.F. Extracellular metabolic energetics can promote cancer progression. *Cell* **2015**, *160*, 393-406, doi:10.1016/j.cell.2014.12.018.
16. Li, X.; Chen, X.; Fu, C.; Xie, M.; Ouyang, S. Long Non-Coding RNA BCAR4 Promotes Oxaliplatin Resistance in Colorectal Cancer by Modulating miR-484-3p/RAB5C Expression. *Chemotherapy* **2023**, *68*, 119-130, doi:10.1159/000529134.
17. Cui, H.; Liu, Y.; Jiang, J.; Liu, Y.; Yang, Z.; Wu, S.; Cao, W.; Cui, I.H.; Yu, C. IGF2-derived miR-483 mediated oncofunction by suppressing DLC-1 and associated with colorectal cancer. *Oncotarget* **2016**, *7*, 48456-48466, doi:10.18632/oncotarget.10309.
18. Sun, L.; Wan, A.; Zhou, Z.; Chen, D.; Liang, H.; Liu, C.; Yan, S.; Niu, Y.; Lin, Z.; Zhan, S.; et al. RNA-binding protein RALY reprogrammes mitochondrial metabolism via mediating miRNA processing in colorectal cancer. *Gut* **2021**, *70*, 1698-1712, doi:10.1136/gutjnl-2020-320652.
19. Veronese, A.; Lupini, L.; Consiglio, J.; Visone, R.; Ferracin, M.; Fornari, F.; Zanesi, N.; Alder, H.; D'Elia, G.; Gramantieri, L.; et al. Oncogenic role of miR-483-3p at the IGF2/483 locus. *Cancer Res* **2010**, *70*, 3140-3149, doi:10.1158/0008-5472.CAN-09-4456.
20. Candiello, E.; Reato, G.; Verginelli, F.; Gambardella, G.; A, D.A.; Calandra, N.; Orzan, F.; Iuliano, A.; Albano, R.; Sassi, F.; et al. MicroRNA 483-3p overexpression unleashes invasive growth of metastatic colorectal cancer via NDRG1 downregulation and ensuing activation of the ERBB3/AKT axis. *Mol Oncol* **2023**, *17*, 1280-1301, doi:10.1002/1878-0261.13408.
21. Song, Q.; Xu, Y.; Yang, C.; Chen, Z.; Jia, C.; Chen, J.; Zhang, Y.; Lai, P.; Fan, X.; Zhou, X.; et al. miR-483-5p promotes invasion and metastasis of lung adenocarcinoma by targeting RhoGDI1 and ALCAM. *Cancer Res* **2014**, *74*, 3031-3042, doi:10.1158/0008-5472.CAN-13-2193.
22. Wang, Y.; Xu, Y.M.; Zou, Y.Q.; Lin, J.; Huang, B.; Liu, J.; Li, J.; Zhang, J.; Yang, W.M.; Min, Q.H.; et al. Identification of differential expressed PE exosomal miRNA in lung adenocarcinoma, tuberculosis, and other benign lesions. *Medicine (Baltimore)* **2017**, *96*, e8361, doi:10.1097/MD.0000000000008361.
23. Wang, F.; Zhang, X.; Zhong, X.; Zhang, M.; Guo, M.; Yang, L.; Li, Y.; Zhao, J.; Yu, S. Effect of miR-483-5p on apoptosis of lung cancer cells through targeting of RBM5. *Int J Clin Exp Pathol* **2018**, *11*, 3147-3156.
24. Yue, J.; Lv, D.; Wang, C.; Li, L.; Zhao, Q.; Chen, H.; Xu, L. Epigenetic silencing of miR-483-3p promotes acquired gefitinib resistance and EMT in EGFR-mutant NSCLC by targeting integrin beta3. *Oncogene* **2018**, *37*, 4300-4312, doi:10.1038/s41388-018-0276-2.
25. Li, S.; Zheng, K.; Pei, Y.; Wang, W.; Zhang, X. Long noncoding RNA NR2F1-AS1 enhances the malignant properties of osteosarcoma by increasing forkhead box A1 expression via sponging of microRNA-483-3p. *Aging (Albany NY)* **2019**, *11*, 11609-11623, doi:10.18632/aging.102563.

26. Chen, Y.; Li, J.; Xiao, J.K.; Xiao, L.; Xu, B.W.; Li, C. The lncRNA NEAT1 promotes the epithelial-mesenchymal transition and metastasis of osteosarcoma cells by sponging miR-483 to upregulate STAT3 expression. *Cancer Cell Int* **2021**, *21*, 90, doi:10.1186/s12935-021-01780-8.
27. Wang, W.; Zhao, L.J.; Yang, Y.; Wang, R.Y.; Ren, H.; Zhao, P.; Zhou, W.P.; Qi, Z.T. Retinoic acid induced 16 enhances tumorigenesis and serves as a novel tumor marker for hepatocellular carcinoma. *Carcinogenesis* **2012**, *33*, 2578-2585, doi:10.1093/carcin/bgs289.
28. Niture, S.; Gadi, S.; Qi, Q.; Gyamfi, M.A.; Varghese, R.S.; Rios-Colon, L.; Chimeh, U.; Vandana; Ressom, H.W.; Kumar, D. MicroRNA-483-5p Inhibits Hepatocellular Carcinoma Cell Proliferation, Cell Steatosis, and Fibrosis by Targeting PPARalpha and TIMP2. *Cancers (Basel)* **2023**, *15*, doi:10.3390/cancers15061715.
29. Ma, N.; Li, F.; Li, D.; Hui, Y.; Wang, X.; Qiao, Y.; Zhang, Y.; Xiang, Y.; Zhou, J.; Zhou, L.; et al. Igf2-derived intronic miR-483 promotes mouse hepatocellular carcinoma cell proliferation. *Mol Cell Biochem* **2012**, *361*, 337-343, doi:10.1007/s11010-011-1121-x.
30. Lu, X.Y.; Chen, D.; Gu, X.Y.; Ding, J.; Zhao, Y.J.; Zhao, Q.; Yao, M.; Chen, Z.; He, X.H.; Cong, W.M. Predicting Value of ALCAM as a Target Gene of microRNA-483-5p in Patients with Early Recurrence in Hepatocellular Carcinoma. *Front Pharmacol* **2017**, *8*, 973, doi:10.3389/fphar.2017.00973.
31. Tang, S.; Chen, Y.; Feng, S.; Yi, T.; Liu, X.; Li, Q.; Liu, Z.; Zhu, C.; Hu, J.; Yu, X.; et al. MiR-483-5p promotes IGF-II transcription and is associated with poor prognosis of hepatocellular carcinoma. *Oncotarget* **2017**, *8*, 99871-99888, doi:10.18632/oncotarget.21737.
32. Zhang, Z.; Ge, S.; Wang, X.; Yuan, Q.; Yan, Q.; Ye, H.; Che, Y.; Lin, Y.; Zhang, J.; Liu, P. Serum miR-483-5p as a potential biomarker to detect hepatocellular carcinoma. *Hepatol Int* **2013**, *7*, 199-207, doi:10.1007/s12072-012-9341-z.
33. Pepe, F.; Pagotto, S.; Soliman, S.; Rossi, C.; Lanuti, P.; Braconi, C.; Mariani-Costantini, R.; Visone, R.; Veronese, A. Regulation of miR-483-3p by the O-linked N-acetylglucosamine transferase links chemosensitivity to glucose metabolism in liver cancer cells. *Oncogenesis* **2017**, *6*, e328, doi:10.1038/oncsis.2017.35.
34. Li, X.; Cheng, T.; He, Y.; Zhou, S.; Wang, Y.; Zhang, K.; Yu, P. High glucose regulates ERp29 in hepatocellular carcinoma by LncRNA MEG3-miRNA 483-3p pathway. *Life Sci* **2019**, *232*, 116602, doi:10.1016/j.lfs.2019.116602.
35. Cui, K.; Zhang, H.; Wang, G.Z. MiR-483 suppresses cell proliferation and promotes cell apoptosis by targeting SOX3 in breast cancer. *Eur Rev Med Pharmacol Sci* **2019**, *23*, 2069-2074, doi:10.26355/eurrev_201903_17248.
36. Huang, X.; Lyu, J. Tumor suppressor function of miR-483-3p on breast cancer via targeting of the cyclin E1 gene. *Exp Ther Med* **2018**, *16*, 2615-2620, doi:10.3892/etm.2018.6504.
37. Cheng, L.; Zhang, X.; Huang, Y.Z.; Zhu, Y.L.; Xu, L.Y.; Li, Z.; Dai, X.Y.; Shi, L.; Zhou, X.J.; Wei, J.F.; et al. Metformin exhibits antiproliferation activity in breast cancer via miR-483-3p/METTL3/m(6)A/p21 pathway. *Oncogenesis* **2021**, *10*, 7, doi:10.1038/s41389-020-00290-y.
38. Menbari, M.N.; Rahimi, K.; Ahmadi, A.; Mohammadi-Yeganeh, S.; Elyasi, A.; Darvishi, N.; Hosseini, V.; Abdi, M. miR-483-3p suppresses the proliferation and progression of human triple negative breast cancer cells by targeting the HDAC8>oncogene. *J Cell Physiol* **2020**, *235*, 2631-2642, doi:10.1002/jcp.29167.

39. Lin, W.D.; Chang, C.H.; Pan, J.K.; Lin, F.C.; Chen, Y.C.; Chen, Y.J.; Wang, P.S.; Hong, W.Q.; Chen, S.Y.; Lin, C.H.; et al. A novel long non-coding RNA MIR4500HG003 promotes tumor metastasis through miR-483-3p-MMP9 axis in triple-negative breast cancer. *Cell Death Dis* **2024**, *15*, 310, doi:10.1038/s41419-024-06675-w.
40. Ozata, D.M.; Caramuta, S.; Velazquez-Fernandez, D.; Akcakaya, P.; Xie, H.; Hoog, A.; Zedenius, J.; Backdahl, M.; Larsson, C.; Lui, W.O. The role of microRNA deregulation in the pathogenesis of adrenocortical carcinoma. *Endocr Relat Cancer* **2011**, *18*, 643-655, doi:10.1530/ERC-11-0082.
41. Agosta, C.; Laugier, J.; Guyon, L.; Denis, J.; Bertherat, J.; Libe, R.; Boisson, B.; Sturm, N.; Feige, J.J.; Chabre, O.; et al. MiR-483-5p and miR-139-5p promote aggressiveness by targeting N-myc downstream-regulated gene family members in adrenocortical cancer. *Int J Cancer* **2018**, *143*, 944-957, doi:10.1002/ijc.31363.
42. Zhang, X.; Liu, L.; Deng, X.; Li, D.; Cai, H.; Ma, Y.; Jia, C.; Wu, B.; Fan, Y.; Lv, Z. MicroRNA 483-3p targets Pard3 to potentiate TGF-beta1-induced cell migration, invasion, and epithelial-mesenchymal transition in anaplastic thyroid cancer cells. *Oncogene* **2019**, *38*, 699-715, doi:10.1038/s41388-018-0447-1.
43. Wu, K.; Wang, J.; He, J.; Chen, Q.; Yang, L. miR-483-3p promotes proliferation and migration of neuroblastoma cells by targeting PUMA. *Int J Clin Exp Pathol* **2018**, *11*, 490-501.
44. Lu, S.; Yu, Z.; Zhang, X.; Sui, L. MiR-483 Targeted SOX3 to Suppress Glioma Cell Migration, Invasion and Promote Cell Apoptosis. *Onco Targets Ther* **2020**, *13*, 2153-2161, doi:10.2147/OTT.S240619.
45. Wang, L.; Shi, M.; Hou, S.; Ding, B.; Liu, L.; Ji, X.; Zhang, J.; Deng, Y. MiR-483-5p suppresses the proliferation of glioma cells via directly targeting ERK1. *FEBS Lett* **2012**, *586*, 1312-1317, doi:10.1016/j.febslet.2012.03.035.
46. Yuan, L.; Zhang, P.; Lu, Y.; Zhang, A.; Chen, X. LINC00662 Promotes Proliferation and Invasion and Inhibits Apoptosis of Glioma Cells Through miR-483-3p/SOX3 Axis. *Appl Biochem Biotechnol* **2022**, doi:10.1007/s12010-022-03855-2.
47. Uhlmann, E.J.; Mackel, C.E.; Deforzh, E.; Rabinovsky, R.; Brastianos, P.K.; Varma, H.; Vega, R.A.; Krichevsky, A.M. Inhibition of the epigenetically activated miR-483-5p/IGF-2 pathway results in rapid loss of meningioma tumor cell viability. *J Neurooncol* **2023**, *162*, 109-118, doi:10.1007/s11060-023-04264-z.
48. Tian, Y.; Yan, M.; Zheng, J.; Li, R.; Lin, J.; Xu, A.; Liang, Y.; Zheng, R.; Yuan, Y. miR-483-5p decreases the radiosensitivity of nasopharyngeal carcinoma cells by targeting DAPK1. *Lab Invest* **2019**, *99*, 602-611, doi:10.1038/s41374-018-0169-6.
49. Li, X.Z.; Tu, Y.J.; Zhou, T.; Zhang, J.B.; Xiao, R.W.; Yang, D.W.; Zhang, P.F.; You, P.T.; Zheng, X.H. MicroRNA-483-5p Predicts Poor Prognosis and Promotes Cancer Metastasis by Targeting EGR3 in Nasopharyngeal Carcinoma. *Front Oncol* **2021**, *11*, 720835, doi:10.3389/fonc.2021.720835.
50. Chen, Y.; Wang, H.; Zhu, S.; Lan, X. miR-483-5p promotes esophageal cancer progression by targeting KCNQ1. *Biochem Biophys Res Commun* **2020**, *531*, 615-621, doi:10.1016/j.bbrc.2020.07.037.
51. Sun, J.; Li, X.; Wang, W.; Li, W.; Gao, S.; Yan, J. Mir-483-5p promotes the malignant transformation of immortalized human esophageal epithelial cells by targeting HNF4A. *Int J Clin Exp Pathol* **2017**, *10*, 9391-9399.

52. Duan, L.; Ma, J.; Yang, W.; Cao, L.; Wang, X.; Niu, L.; Li, Y.; Zhou, W.; Zhang, Y.; Liu, J.; et al. EI24 Inhibits Cell Proliferation and Drug Resistance of Esophageal Squamous Cell Carcinoma. *Front Oncol* **2020**, *10*, 1570, doi:10.3389/fonc.2020.01570.
53. Ma, J.; Hong, L.; Xu, G.; Hao, J.; Wang, R.; Guo, H.; Liu, J.; Zhang, Y.; Nie, Y.; Fan, D. miR-483-3p plays an oncogenic role in esophageal squamous cell carcinoma by targeting tumor suppressor EI24. *Cell Biol Int* **2016**, *40*, 448-455, doi:10.1002/cbin.10585.
54. Yang, Z.G.; Ma, X.D.; He, Z.H.; Guo, Y.X. miR-483-5p promotes prostate cancer cell proliferation and invasion by targeting RBM5. *Int Braz J Urol* **2017**, *43*, 1060-1067, doi:10.1590/S1677-5538.IBJU.2016.0595.
55. Fan, L.; Li, H.; Zhang, Y. LINC00908 negatively regulates microRNA-483-5p to increase TSPYL5 expression and inhibit the development of prostate cancer. *Cancer Cell Int* **2020**, *20*, 10, doi:10.1186/s12935-019-1073-x.
56. Chow, J.T.; Desjardins, A.; Lee, D.K.C.; Grigore, I.A.; Lee, L.; Fu, N.J.; Chau, S.; Lee, B.Y.; Gabra, M.M.; Salmena, L. A microRNA CRISPR screen reveals microRNA-483-3p as an apoptotic regulator in prostate cancer cells. *Cell Death Dis* **2025**, *16*, 752, doi:10.1038/s41419-025-08098-7.
57. Arrighetti, N.; Cossa, G.; De Cecco, L.; Stucchi, S.; Carenini, N.; Corna, E.; Gandellini, P.; Zaffaroni, N.; Perego, P.; Gatti, L. PKC-alpha modulation by miR-483-3p in platinum-resistant ovarian carcinoma cells. *Toxicol Appl Pharmacol* **2016**, *310*, 9-19, doi:10.1016/j.taap.2016.08.005.
58. Rattanapan, Y.; Korkiatsakul, V.; Kongruang, A.; Siriboonpiputtana, T.; Rerkamnuaychoke, B.; Chareonsirisuthigul, T. High Expression of miR-483-5p Predicts Chemotherapy Resistance in Epithelial Ovarian Cancer. *Microrna* **2021**, *10*, 51-57, doi:10.2174/2211536610666210412155206.
59. Hao, J.; Zhang, S.; Zhou, Y.; Hu, X.; Shao, C. MicroRNA 483-3p suppresses the expression of DPC4/Smad4 in pancreatic cancer. *FEBS Lett* **2011**, *585*, 207-213, doi:10.1016/j.febslet.2010.11.039.
60. Shao, H.; Zhang, Y.; Yan, J.; Ban, X.; Fan, X.; Chang, X.; Lu, Z.; Wu, Y.; Zong, L.; Mo, S.; et al. Upregulated MicroRNA-483-3p is an Early Event in Pancreatic Ductal Adenocarcinoma (PDAC) and as a Powerful Liquid Biopsy Biomarker in PDAC. *Onco Targets Ther* **2021**, *14*, 2163-2175, doi:10.2147/OTT.S288936.
61. Zhang, L.; Ruan, Y.; Qin, Z.; Gao, X.; Xu, K.; Shi, X.; Gao, S.; Liu, S.; Zhu, K.; Wang, W.; et al. miR-483-3p, Mediated by KLF9, Functions as Tumor Suppressor in Testicular Seminoma via Targeting MMP9. *Front Oncol* **2020**, *10*, 596574, doi:10.3389/fonc.2020.596574.
62. Castro-Vega, L.J.; Calsina, B.; Burnichon, N.; Drossart, T.; Martinez-Montes, A.M.; Verkarre, V.; Amar, L.; Bertherat, J.; Rodriguez-Antona, C.; Favier, J.; et al. Overexpression of miR-483-5p is confined to metastases and linked to high circulating levels in patients with metastatic pheochromocytoma/paraganglioma. *Clin Transl Med* **2020**, *10*, e260, doi:10.1002/ctm2.260.
63. Wu, W.; Wei, N.; Shao, G.; Jiang, C.; Zhang, S.; Wang, L. circZNF609 promotes the proliferation and migration of gastric cancer by sponging miR-483-3p and regulating CDK6. *Onco Targets Ther* **2019**, *12*, 8197-8205, doi:10.2147/OTT.S193031.
64. Yu, F.Y.; Zhou, C.Y.; Liu, Y.B.; Wang, B.; Mao, L.; Li, Y. miR-483 is down-regulated in gastric cancer and suppresses cell proliferation, invasion and protein O-GlcNAcylation by targeting OGT. *Neoplasma* **2018**, *65*, 406-414, doi:10.4149/neo_2018_170608N411.
65. Wu, Q.; Ma, J.; Wei, J.; Meng, W.; Wang, Y.; Shi, M. lncRNA SNHG11 Promotes Gastric Cancer Progression by Activating the Wnt/beta-Catenin Pathway and Oncogenic Autophagy. *Mol Ther* **2021**, *29*, 1258-1278, doi:10.1016/j.ymthe.2020.10.011.

66. Liu, K.; He, B.; Xu, J.; Li, Y.; Guo, C.; Cai, Q.; Wang, S. miR-483-5p Targets MKNK1 to Suppress Wilms' Tumor Cell Proliferation and Apoptosis In Vitro and In Vivo. *Med Sci Monit* **2019**, *25*, 1459-1468, doi:10.12659/MSM.913005.
67. Che, G.; Gao, H.; Tian, J.; Hu, Q.; Xie, H.; Zhang, Y. MicroRNA-483-3p Promotes Proliferation, Migration, and Invasion and Induces Chemoresistance of Wilms' Tumor Cells. *Pediatr Dev Pathol* **2020**, *23*, 144-151, doi:10.1177/1093526619873491.
68. Liu, M.; Roth, A.; Yu, M.; Morris, R.; Bersani, F.; Rivera, M.N.; Lu, J.; Shioda, T.; Vasudevan, S.; Ramaswamy, S.; et al. The IGF2 intronic miR-483 selectively enhances transcription from IGF2 fetal promoters and enhances tumorigenesis. *Genes Dev* **2013**, *27*, 2543-2548, doi:10.1101/gad.224170.113.
69. Guled, M.; Lahti, L.; Lindholm, P.M.; Salmenkivi, K.; Bagwan, I.; Nicholson, A.G.; Knuutila, S. CDKN2A, NF2, and JUN are dysregulated among other genes by miRNAs in malignant mesothelioma -A miRNA microarray analysis. *Genes Chromosomes Cancer* **2009**, *48*, 615-623, doi:10.1002/gcc.20669.
70. Feng, X.; Yang, L.; Liu, X.; Liu, M.; Liu, L.; Liu, J.; Luo, J. Long non-coding RNA small nucleolar RNA host gene 29 drives chronic myeloid leukemia progression via microRNA-483-3p/Casitas B-lineage Lymphoma axis-mediated activation of the phosphoinositide 3-kinase/Akt pathway. *Med Oncol* **2024**, *41*, 60, doi:10.1007/s12032-023-02287-0.
71. Bertero, T.; Bourget-Ponzio, I.; Puissant, A.; Loubat, A.; Mari, B.; Meneguzzi, G.; Auberger, P.; Barbry, P.; Ponzio, G.; Rezzonico, R. Tumor suppressor function of miR-483-3p on squamous cell carcinomas due to its pro-apoptotic properties. *Cell Cycle* **2013**, *12*, 2183-2193, doi:10.4161/cc.25330.
72. Fan, S.; Chen, W.X.; Lv, X.B.; Tang, Q.L.; Sun, L.J.; Liu, B.D.; Zhong, J.L.; Lin, Z.Y.; Wang, Y.Y.; Li, Q.X.; et al. miR-483-5p determines mitochondrial fission and cisplatin sensitivity in tongue squamous cell carcinoma by targeting FIS1. *Cancer Lett* **2015**, *362*, 183-191, doi:10.1016/j.canlet.2015.03.045.
73. Gu, J.; Wang, M.; Wang, X.; Li, J.; Liu, H.; Lin, Z.; Yang, X.; Zhang, X.; Liu, H. Exosomal miR-483-5p in Bone Marrow Mesenchymal Stem Cells Promotes Malignant Progression of Multiple Myeloma by Targeting TIMP2. *Front Cell Dev Biol* **2022**, *10*, 862524, doi:10.3389/fcell.2022.862524.
74. Maemura, T.; Fukuyama, S.; Sugita, Y.; Lopes, T.J.S.; Nakao, T.; Noda, T.; Kawaoka, Y. Lung-Derived Exosomal miR-483-3p Regulates the Innate Immune Response to Influenza Virus Infection. *J Infect Dis* **2018**, *217*, 1372-1382, doi:10.1093/infdis/jiy035.
75. Wang, Z.; Qin, X.; Yuan, J.; Yin, H.; Qu, R.; Zhong, C.; Ding, W. MicroRNA-483-3p Inhibitor Ameliorates Sepsis-Induced Intestinal Injury by Attenuating Cell Apoptosis and Cytotoxicity Via Regulating HIPK2. *Mol Biotechnol* **2024**, *66*, 233-240, doi:10.1007/s12033-023-00734-x.
76. Zhou, J.; Lin, J.; Zhao, Y.; Sun, X. Deregulated Expression of miR-483-3p Serves as a Diagnostic Biomarker in Severe Pneumonia Children with Respiratory Failure and Its Predictive Value for the Clinical Outcome of Patients. *Mol Biotechnol* **2022**, *64*, 311-319, doi:10.1007/s12033-021-00415-7.
77. Chouri, E.; Servaas, N.H.; Bekker, C.P.J.; Affandi, A.J.; Cossu, M.; Hillen, M.R.; Angiolilli, C.; Mertens, J.S.; van den Hoogen, L.L.; Silva-Cardoso, S.; et al. Serum microRNA screening and functional studies reveal miR-483-5p as a potential driver of fibrosis in systemic sclerosis. *J Autoimmun* **2018**, *89*, 162-170, doi:10.1016/j.jaut.2017.12.015.
78. Zhang, C.; Gao, C.; Di, X.; Cui, S.; Liang, W.; Sun, W.; Yao, M.; Liu, S.; Zheng, Z. Hsa_circ_0123190 acts as a competitive endogenous RNA to regulate APLNR expression by sponging hsa-miR-483-3p in lupus nephritis. *Arthritis Res Ther* **2021**, *23*, 24, doi:10.1186/s13075-020-02404-8.
79. Li, J.; Chen, M.; Wang, J.; Lu, L.; Li, X.; Le, Y. MicroRNA profiling in Chinese children with Henoch-Schonlein purpura and association between selected microRNAs and inflammatory biomarkers. *Acta Paediatr* **2021**, *110*, 2221-2229, doi:10.1111/apa.15789.

80. Yu, Y.; Park, S.; Lee, H.; Kwon, E.J.; Park, H.R.; Kim, Y.H.; Lee, S.G. Exosomal hsa-miR-335-5p and hsa-miR-483-5p are novel biomarkers for rheumatoid arthritis: A development and validation study. *Int Immunopharmacol* **2023**, *120*, 110286, doi:10.1016/j.intimp.2023.110286.
81. Liu, X.; Guo, L.; Du, J.; Luo, Z.; Xu, J.; Bhawal, U.K.; Li, X.; Liu, Y. Macrophage-derived apoptotic bodies impair the osteogenic ability of osteoblasts in periodontitis. *Oral Dis* **2024**, *30*, 3296-3307, doi:10.1111/odi.14808.
82. Xiong, Y.; Chen, X.; Yang, X.; Zhang, H.; Li, X.; Wang, Z.; Feng, S.; Wen, W.; Xiong, X. miRNA transcriptomics analysis shows miR-483-5p and miR-503-5p targeted miRNA in extracellular vesicles from severe acute pancreatitis-associated lung injury patients. *Int Immunopharmacol* **2023**, *125*, 111075, doi:10.1016/j.intimp.2023.111075.
83. He, M.; Chen, Z.; Martin, M.; Zhang, J.; Sangwung, P.; Woo, B.; Tremoulet, A.H.; Shimizu, C.; Jain, M.K.; Burns, J.C.; et al. miR-483 Targeting of CTGF Suppresses Endothelial-to-Mesenchymal Transition: Therapeutic Implications in Kawasaki Disease. *Circ Res* **2017**, *120*, 354-365, doi:10.1161/CIRCRESAHA.116.310233.
84. Ferland-McCollough, D.; Fernandez-Twinn, D.S.; Cannell, I.G.; David, H.; Warner, M.; Vaag, A.A.; Bork-Jensen, J.; Brons, C.; Gant, T.W.; Willis, A.E.; et al. Programming of adipose tissue miR-483-3p and GDF-3 expression by maternal diet in type 2 diabetes. *Cell Death Differ* **2012**, *19*, 1003-1012, doi:10.1038/cdd.2011.183.
85. Wang, Z.; Mohan, R.; Chen, X.; Matson, K.; Waugh, J.; Mao, Y.; Zhang, S.; Li, W.; Tang, X.; Satin, L.S.; et al. microRNA-483 Protects Pancreatic beta-Cells by Targeting ALDH1A3. *Endocrinology* **2021**, *162*, doi:10.1210/endocr/bqab031.
86. Yuan, H.; He, M.; Yang, Q.; Niu, F.; Zou, Y.; Liu, C.; Yang, Y.; Liu, A.; Chang, X.; Chen, F.; et al. Obesity-induced upregulation of miR-483-5p impairs the function and identity of pancreatic beta-cells. *Diabetes Obes Metab* **2024**, *26*, 4510-4521, doi:10.1111/dom.15805.
87. Liu, L.; Chen, H.; Yun, J.; Song, L.; Ma, X.; Luo, S.; Song, Y. miRNA-483-5p Targets HDCA4 to Regulate Renal Tubular Damage in Diabetic Nephropathy. *Horm Metab Res* **2021**, *53*, 562-569, doi:10.1055/a-1480-7519.
88. Liu, D.; Liu, F.; Li, Z.; Pan, S.; Xie, J.; Zhao, Z.; Liu, Z.; Zhang, J.; Liu, Z. HNRNPA1-mediated exosomal sorting of miR-483-5p out of renal tubular epithelial cells promotes the progression of diabetic nephropathy-induced renal interstitial fibrosis. *Cell Death Dis* **2021**, *12*, 255, doi:10.1038/s41419-021-03460-x.
89. Abudoureyimu, M.; Tayier, T.; Zhang, L. The role and mechanism of action of miR-483-3p in mediating the effects of IGF-1 on human renal tubular epithelial cells induced by high glucose. *Sci Rep* **2024**, *14*, 15635, doi:10.1038/s41598-024-66433-y.
90. Dong, J.; He, M.; Li, J.; Pessentheiner, A.; Wang, C.; Zhang, J.; Sun, Y.; Wang, W.T.; Zhang, Y.; Liu, J.; et al. microRNA-483 ameliorates hypercholesterolemia by inhibiting PCSK9 production. *JCI Insight* **2020**, *5*, doi:10.1172/jci.insight.143812.
91. Liu, H.; French, B.A.; Li, J.; Tillman, B.; French, S.W. Altered regulation of miR-34a and miR-483-3p in alcoholic hepatitis and DDC fed mice. *Exp Mol Pathol* **2015**, *99*, 552-557, doi:10.1016/j.yexmp.2015.09.005.
92. Cao, D.; Zhou, L.; Hu, R. Exosomes derived from BMSCs alleviates high glucose-induced diabetic retinopathy via carrying miR-483-5p. *J Biochem Mol Toxicol* **2024**, *38*, e23616, doi:10.1002/jbt.23616.

93. Kemp, J.R.; Unal, H.; Desnoyer, R.; Yue, H.; Bhatnagar, A.; Karnik, S.S. Angiotensin II-regulated microRNA 483-3p directly targets multiple components of the renin-angiotensin system. *J Mol Cell Cardiol* **2014**, *75*, 25-39, doi:10.1016/j.yjmcc.2014.06.008.
94. Zhang, J.; He, Y.; Yan, X.; Chen, S.; He, M.; Lei, Y.; Zhang, J.; Gongol, B.; Gu, M.; Miao, Y.; et al. MicroRNA-483 amelioration of experimental pulmonary hypertension. *EMBO Mol Med* **2020**, *12*, e11303, doi:10.15252/emmm.201911303.
95. Zhu, H.; Liang, H.; Gao, Z.; Zhang, X.; He, Q.; He, C.; Cai, C.; Chen, J. MiR-483-5p downregulation alleviates ox-LDL induced endothelial cell injury in atherosclerosis. *BMC Cardiovasc Disord* **2023**, *23*, 521, doi:10.1186/s12872-023-03496-1.
96. Fernandez Esmerats, J.; Villa-Roel, N.; Kumar, S.; Gu, L.; Salim, M.T.; Ohh, M.; Taylor, W.R.; Nerem, R.M.; Yoganathan, A.P.; Jo, H. Disturbed Flow Increases UBE2C (Ubiquitin E2 Ligase C) via Loss of miR-483-3p, Inducing Aortic Valve Calcification by the pVHL (von Hippel-Lindau Protein) and HIF-1alpha (Hypoxia-Inducible Factor-1alpha) Pathway in Endothelial Cells. *Arterioscler Thromb Vasc Biol* **2019**, *39*, 467-481, doi:10.1161/ATVBAHA.118.312233.
97. Kong, L.; Hu, N.; Du, X.; Wang, W.; Chen, H.; Li, W.; Wei, S.; Zhuang, H.; Li, X.; Li, C. Upregulation of miR-483-3p contributes to endothelial progenitor cells dysfunction in deep vein thrombosis patients via SRF. *J Transl Med* **2016**, *14*, 23, doi:10.1186/s12967-016-0775-2.
98. Fan, J.; Liu, S.; Ye, W.; Zhang, X.; Shi, W. miR-483-5p-Containing exosomes treatment ameliorated deep vein thrombosis-induced inflammatory response. *Eur J Pharm Biopharm* **2024**, *202*, 114384, doi:10.1016/j.ejpb.2024.114384.
99. Qiao, Y.; Zhao, Y.; Liu, Y.; Ma, N.; Wang, C.; Zou, J.; Liu, Z.; Zhou, Z.; Han, D.; He, J.; et al. miR-483-3p regulates hyperglycaemia-induced cardiomyocyte apoptosis in transgenic mice. *Biochem Biophys Res Commun* **2016**, *477*, 541-547, doi:10.1016/j.bbrc.2016.06.051.
100. Qiao, Y.; Ma, N.; Wang, X.; Hui, Y.; Li, F.; Xiang, Y.; Zhou, J.; Zou, C.; Jin, J.; Lv, G.; et al. MiR-483-5p controls angiogenesis in vitro and targets serum response factor. *FEBS Lett* **2011**, *585*, 3095-3100, doi:10.1016/j.febslet.2011.08.039.
101. Saddic, L.A.; Chang, T.W.; Sigurdsson, M.I.; Heydarpour, M.; Raby, B.A.; Shernan, S.K.; Aranki, S.F.; Body, S.C.; Muehlschlegel, J.D. Integrated microRNA and mRNA responses to acute human left ventricular ischemia. *Physiol Genomics* **2015**, *47*, 455-462, doi:10.1152/physiolgenomics.00049.2015.
102. Sun, H.; Cai, J.; Xu, L.; Liu, J.; Chen, M.; Zheng, M.; Wang, L.; Yang, X. miR-483-3p regulates acute myocardial infarction by transcriptionally repressing insulin growth factor 1 expression. *Mol Med Rep* **2018**, *17*, 4785-4790, doi:10.3892/mmr.2018.8456.
103. Kuschnerus, K.; Straessler, E.T.; Muller, M.F.; Luscher, T.F.; Landmesser, U.; Krankel, N. Increased Expression of miR-483-3p Impairs the Vascular Response to Injury in Type 2 Diabetes. *Diabetes* **2019**, *68*, 349-360, doi:10.2337/db18-0084.
104. Zhang, Q.; Zhan, H.; Liu, C.; Zhang, C.; Wei, H.; Li, B.; Zhou, D.; Lu, Y.; Huang, S.; Cheng, J.; et al. Neuroprotective Effect of miR-483-5p Against Cardiac Arrest-Induced Mitochondrial Dysfunction Mediated Through the TNFSF8/AMPK/JNK Signaling Pathway. *Cell Mol Neurobiol* **2023**, *43*, 2179-2202, doi:10.1007/s10571-022-01296-3.
105. Zhang, H.; Wang, J.; Du, A.; Li, Y. MiR-483-3p inhibition ameliorates myocardial ischemia/reperfusion injury by targeting the MDM4/p53 pathway. *Mol Immunol* **2020**, *125*, 9-14, doi:10.1016/j.molimm.2020.06.014.

106. Shen, Z.; Tang, W.; Guo, J.; Sun, S. miR-483-5p plays a protective role in chronic obstructive pulmonary disease. *Int J Mol Med* **2017**, *40*, 193-200, doi:10.3892/ijmm.2017.2996.
107. Leng, C.; Sun, J.; Xin, K.; Ge, J.; Liu, P.; Feng, X. High expression of miR-483-5p aggravates sepsis-induced acute lung injury. *J Toxicol Sci* **2020**, *45*, 77-86, doi:10.2131/jts.45.77.
108. Diaz-Prado, S.; Cicione, C.; Muinos-Lopez, E.; Hermida-Gomez, T.; Oreiro, N.; Fernandez-Lopez, C.; Blanco, F.J. Characterization of microRNA expression profiles in normal and osteoarthritic human chondrocytes. *BMC Musculoskelet Disord* **2012**, *13*, 144, doi:10.1186/1471-2474-13-144.
109. Li, K.; Chen, S.; Cai, P.; Chen, K.; Li, L.; Yang, X.; Yi, J.; Luo, X.; Du, Y.; Zheng, H. MiRNA-483-5p is involved in the pathogenesis of osteoporosis by promoting osteoclast differentiation. *Mol Cell Probes* **2020**, *49*, 101479, doi:10.1016/j.mcp.2019.101479.
110. Zhou, Y.; Jia, H.; Hu, A.; Liu, R.; Zeng, X.; Wang, H. Nanoparticles Targeting Delivery Antagomir-483-5p to Bone Marrow Mesenchymal Stem Cells Treat Osteoporosis by Increasing Bone Formation. *Curr Stem Cell Res Ther* **2023**, *18*, 115-126, doi:10.2174/1574888X17666220426120850.
111. Zhao, F.; Xu, Y.; Ouyang, Y.; Wen, Z.; Zheng, G.; Wan, T.; Sun, G. Silencing of miR-483-5p alleviates postmenopausal osteoporosis by targeting SATB2 and PI3K/AKT pathway. *Aging (Albany NY)* **2021**, *13*, 6945-6956, doi:10.18632/aging.202552.
112. Yang, S.; Li, L.; Zhu, L.; Zhang, C.; Li, Z.; Guo, Y.; Nie, Y.; Luo, Z. Bu-Shen-Huo-Xue-Fang modulates nucleus pulposus cell proliferation and extracellular matrix remodeling in intervertebral disk degeneration through miR-483 regulation of Wnt pathway. *J Cell Biochem* **2019**, *120*, 19318-19329, doi:10.1002/jcb.26760.
113. Wang, C.L.; Zuo, B.; Li; Zhu, J.F.; Xiao, F.; Zhang, X.L.; Chen, X.D. The long noncoding RNA H19 attenuates force-driven cartilage degeneration via miR-483-5p/Dusp5. *Biochem Biophys Res Commun* **2020**, *529*, 210-217, doi:10.1016/j.bbrc.2020.05.180.
114. Wang, Y.; Hou, L.; Yuan, X.; Xu, N.; Zhao, S.; Yang, L.; Zhang, N. miR-483-3p promotes cell proliferation and suppresses apoptosis in rheumatoid arthritis fibroblast-like synoviocytes by targeting IGF-1. *Biomed Pharmacother* **2020**, *130*, 110519, doi:10.1016/j.biopha.2020.110519.
115. Anderson, B.A.; McAlinden, A. miR-483 targets SMAD4 to suppress chondrogenic differentiation of human mesenchymal stem cells. *J Orthop Res* **2017**, *35*, 2369-2377, doi:10.1002/jor.23552.
116. Mayor-Lynn, K.; Toloubeydokhti, T.; Cruz, A.C.; Chegini, N. Expression profile of microRNAs and mRNAs in human placentas from pregnancies complicated by preeclampsia and preterm labor. *Reprod Sci* **2011**, *18*, 46-56, doi:10.1177/1933719110374115.
117. Han, L.; Luo, Q.Q.; Peng, M.G.; Zhang, Y.; Zhu, X.H. miR-483 is downregulated in pre-eclampsia via targeting insulin-like growth factor 1 (IGF1) and regulates the PI3K/Akt/mTOR pathway of endothelial progenitor cells. *J Obstet Gynaecol Res* **2021**, *47*, 63-72, doi:10.1111/jog.14412.
118. Xiang, Y.; Song, Y.; Li, Y.; Zhao, D.; Ma, L.; Tan, L. miR-483 is Down-Regulated in Polycystic Ovarian Syndrome and Inhibits KGN Cell Proliferation via Targeting Insulin-Like Growth Factor 1 (IGF1). *Med Sci Monit* **2016**, *22*, 3383-3393, doi:10.12659/msm.897301.
119. Shi, L.; Liu, S.; Zhao, W.; Shi, J. miR-483-5p and miR-486-5p are down-regulated in cumulus cells of metaphase II oocytes from women with polycystic ovary syndrome. *Reprod Biomed Online* **2015**, *31*, 565-572, doi:10.1016/j.rbmo.2015.06.023.
120. Xu, B.; Zhang, Y.W.; Tong, X.H.; Liu, Y.S. Characterization of microRNA profile in human cumulus granulosa cells: Identification of microRNAs that regulate Notch signaling and are associated with PCOS. *Mol Cell Endocrinol* **2015**, *404*, 26-36, doi:10.1016/j.mce.2015.01.030.

121. Nagaraj, S.; Want, A.; Laskowska-Kaszub, K.; Fesiuk, A.; Vaz, S.; Logarinho, E.; Wojda, U. Candidate Alzheimer's Disease Biomarker miR-483-5p Lowers TAU Phosphorylation by Direct ERK1/2 Repression. *Int J Mol Sci* **2021**, *22*, doi:10.3390/ijms22073653.
122. Luo, G.; Wang, X.; Liu, C. MiR-483-3p improves learning and memory abilities via XPO1 in Alzheimer's disease. *Brain Behav* **2022**, *12*, e2680, doi:10.1002/brb3.2680.
123. Mucha, M.; Skrzypiec, A.E.; Kolenchery, J.B.; Brambilla, V.; Patel, S.; Labrador-Ramos, A.; Kudla, L.; Murrall, K.; Skene, N.; Dymicka-Piekarska, V.; et al. miR-483-5p offsets functional and behavioural effects of stress in male mice through synapse-targeted repression of Pgap2 in the basolateral amygdala. *Nat Commun* **2023**, *14*, 2134, doi:10.1038/s41467-023-37688-2.
124. Coenen-Stass, A.M.L.; Sork, H.; Gatto, S.; Godfrey, C.; Bhomra, A.; Krjutskov, K.; Hart, J.R.; Westholm, J.O.; O'Donovan, L.; Roos, A.; et al. Comprehensive RNA-Sequencing Analysis in Serum and Muscle Reveals Novel Small RNA Signatures with Biomarker Potential for DMD. *Mol Ther Nucleic Acids* **2018**, *13*, 1-15, doi:10.1016/j.omtn.2018.08.005.
125. Chen, K.; He, H.; Xie, Y.; Zhao, L.; Zhao, S.; Wan, X.; Yang, W.; Mo, Z. miR-125a-3p and miR-483-5p promote adipogenesis via suppressing the RhoA/ROCK1/ERK1/2 pathway in multiple symmetric lipomatosis. *Sci Rep* **2015**, *5*, 11909, doi:10.1038/srep11909.
126. Zhi, Z.; Zhu, H.; Lv, X.; Lu, C.; Li, Y.; Wu, F.; Zhou, L.; Li, H.; Tang, W. IGF2-derived miR-483-3p associated with Hirschsprung's disease by targeting FHL1. *J Cell Mol Med* **2018**, *22*, 4913-4921, doi:10.1111/jcmm.13756.
127. Wang, G.; Guo, F.; Wang, H.; Liu, W.; Zhang, L.; Cui, M.; Wu, X. Downregulation of microRNA-483-5p Promotes Cell Proliferation and Invasion by Targeting GFRA4 in Hirschsprung's Disease. *DNA Cell Biol* **2017**, *36*, 930-937, doi:10.1089/dna.2017.3821.
