# Supplementary figures and images for: Integrative Regulatory Networks of MicroRNA-483: Unveiling Its Systematic Role in Human Diseases and Clinical Implications
Source: Biomolecules. 2025 Dec 7;15(12):1707. doi: 10.3390/biom15121707 (PMC12730746; doi:10.3390/biom15121707)

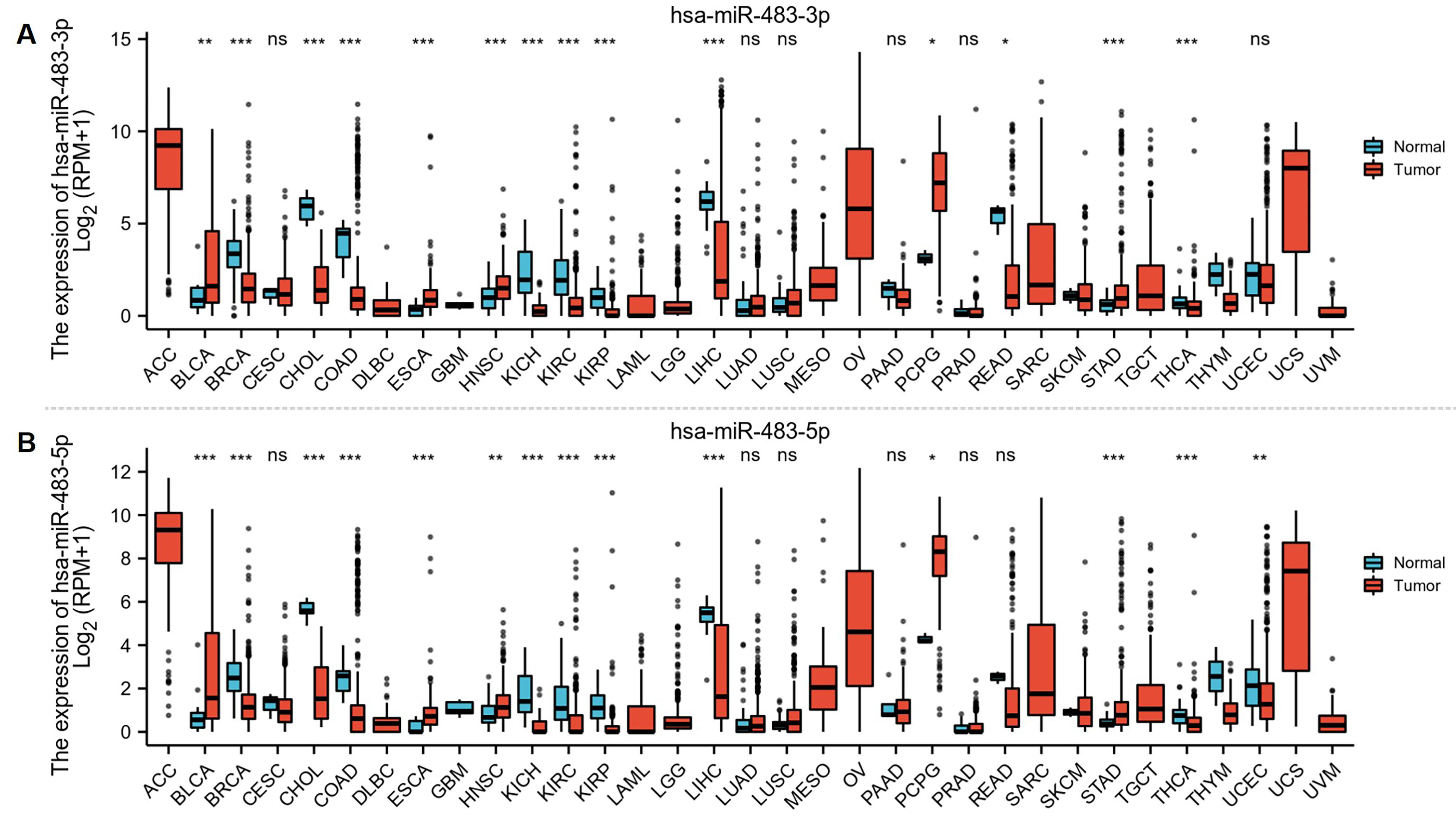

Supplement: Supplementary file 1 [file biomolecules-15-01707-s001.zip › Supplementary Figure S1.jpg]

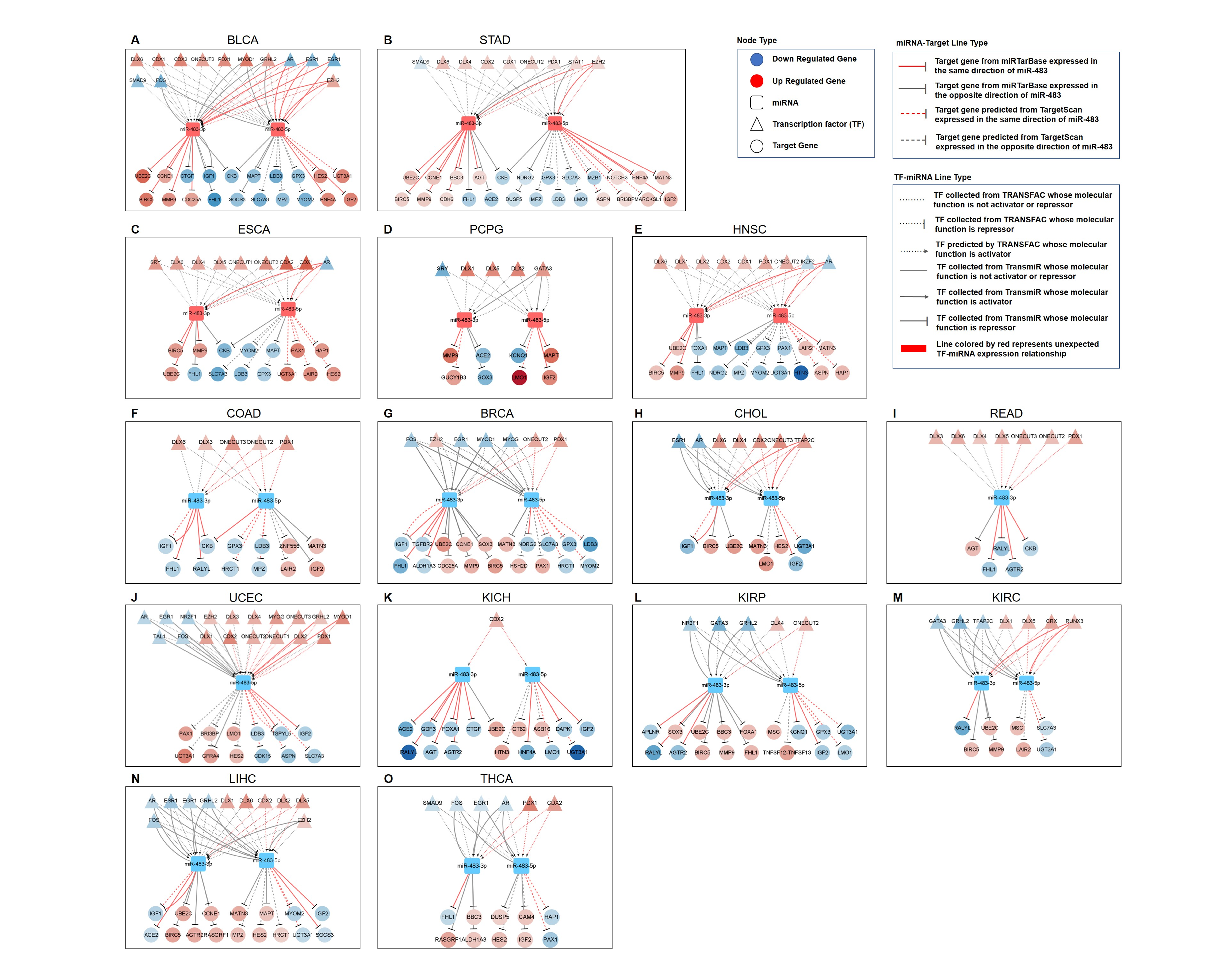

Supplement: Supplementary file 1 [file biomolecules-15-01707-s001.zip › Supplementary Figure S2.png]

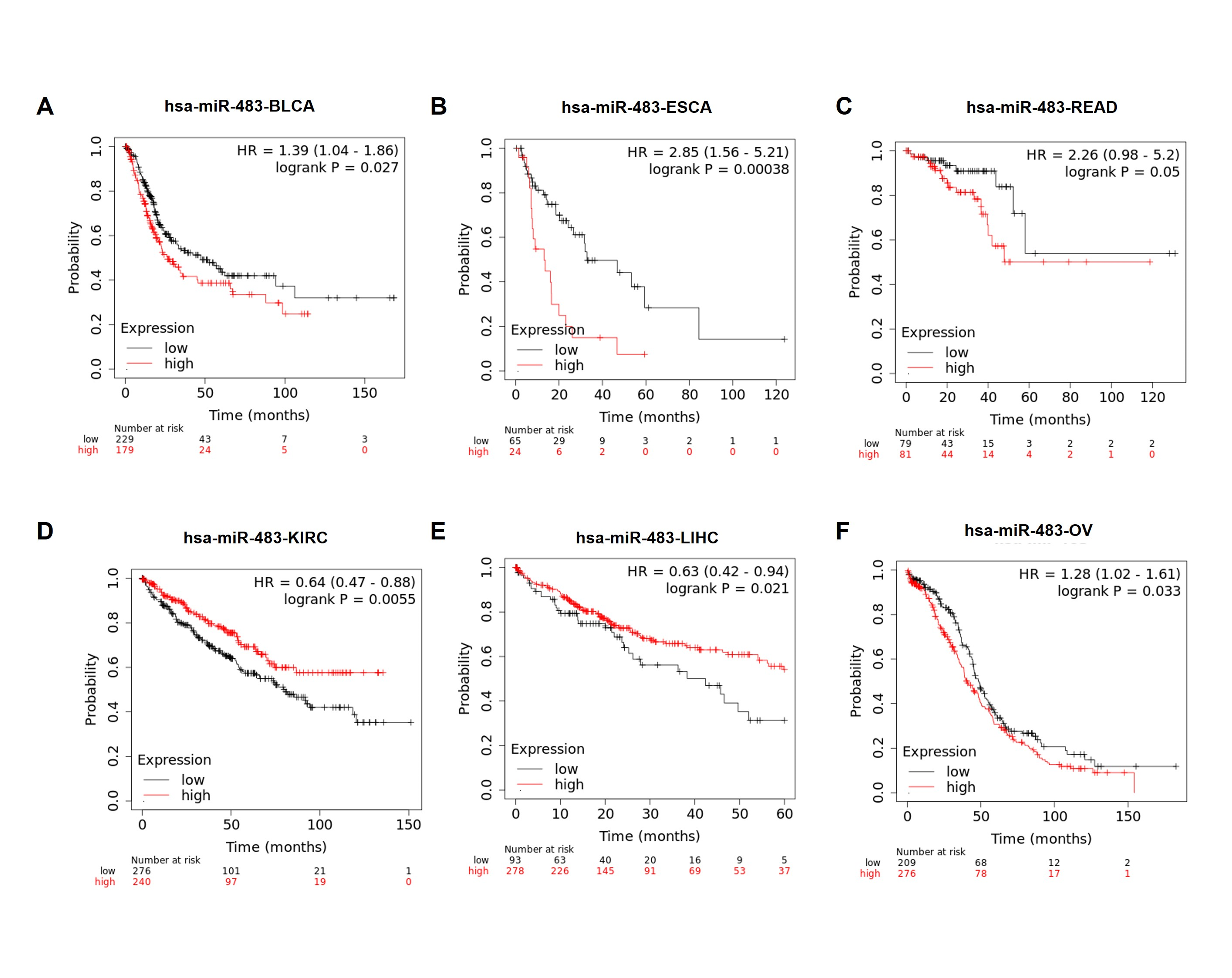

Supplement: Supplementary file 1 [file biomolecules-15-01707-s001.zip › Supplementary Figure S3.png]
